# Saccharomyces cerevisiae: First Steps to a Suitable Model System To Study the Function and Intracellular Transport of Human Kidney Anion Exchanger 1

**DOI:** 10.1128/mSphere.00802-19

**Published:** 2020-01-29

**Authors:** Hasib A. M. Sarder, Xiaobing Li, Charlotta Funaya, Emmanuelle Cordat, Manfred J. Schmitt, Björn Becker

**Affiliations:** a Molecular and Cell Biology, Department of Biosciences (FR 8.3) and Center of Human and Molecular Biology (ZHMB), Saarland University, Saarbrücken, Germany; b Electron Microscopy Core Facility, Heidelberg University, Heidelberg, Germany; c Department of Physiology and Membrane Protein Disease Research Group, University of Alberta, Edmonton, Alberta, Canada; Duke University Medical Center

**Keywords:** *S. cerevisiae*, model organism, kidney anion exchanger 1 (kAE1), heterologous expression, electron microscopy, pH, anion exchange, plasma membrane transport

## Abstract

Saccharomyces cerevisiae has been frequently used to study biogenesis, functionality, and intracellular transport of various renal proteins, including ion channels, solute transporters, and aquaporins. Specific mutations in genes encoding most of these renal proteins affect kidney function in such a way that various disease phenotypes ultimately occur. In this context, human kidney anion exchanger 1 (kAE1) represents an important bicarbonate/chloride exchanger which maintains the acid-base homeostasis in the human body. Malfunctions in kAE1 lead to a pathological phenotype known as distal renal tubular acidosis (dRTA). Here, we evaluated the potential of baker's yeast as a model system to investigate different cellular aspects of kAE1 physiology. For the first time, we successfully expressed yeast codon-optimized full-length versions of tagged and untagged wild-type kAE1 and demonstrated their partial localization at the yeast plasma membrane (PM). Finally, pH and chloride measurements further suggest biological activity of full-length kAE1, emphasizing the potential of S. cerevisiae as a model system for studying trafficking, activity, and/or degradation of mammalian ion channels and transporters such as kAE1 in the future.

**IMPORTANCE** Distal renal tubular acidosis (dRTA) is a common kidney dysfunction characterized by impaired acid secretion via urine. Previous studies revealed that α-intercalated cells of dRTA patients express mutated forms of human kidney anion exchanger 1 (kAE1) which result in inefficient plasma membrane targeting or diminished expression levels of kAE1. However, the precise dRTA-causing processes are inadequately understood, and alternative model systems are helpful tools to address kAE1-related questions in a fast and inexpensive way. In contrast to a previous study, we successfully expressed full-length kAE1 in Saccharomyces cerevisiae. Using advanced microscopy techniques as well as different biochemical and functionality assays, plasma membrane localization and biological activity were confirmed for the heterologously expressed anion transporter. These findings represent first important steps to use the potential of yeast as a model organism for studying trafficking, activity, and degradation of kAE1 and its mutant variants in the future.

## Abstract (precis)

Distal renal tubular acidosis (dRTA) is a common kidney dysfunction characterized by impaired acid secretion via urine. Previous studies revealed that α-intercalated cells of dRTA patients express mutated forms of human kidney anion exchanger 1 (kAE1) which result in inefficient plasma membrane targeting or diminished expression levels of kAE1. However, the precise dRTA-causing processes are inadequately understood, and alternative model systems are helpful tools to address kAE1-related questions in a fast and inexpensive way. In contrast to a previous study, we successfully expressed full-length kAE1 in Saccharomyces cerevisiae. Using advanced microscopy techniques as well as different biochemical and functionality assays, plasma membrane localization and biological activity were confirmed for the heterologously expressed anion transporter. These findings represent first important steps to use the potential of yeast as a model organism for studying trafficking, activity, and degradation of kAE1 and its mutant variants in the future.

## INTRODUCTION

As central organs of the urinary system, kidneys are essential for water-electrolyte and acid-base homeostasis. During urine formation, different types of intercalated cells (IC) in the connecting tubule and the collecting duct are responsible for the fine-tuning of urine composition and urine acidification (1). Depending on the expression and localization profile of V-H^+^-ATPase, kidney anion exchanger 1 (kAE1), and the anion transporter pendrin, intercalated cells are classified as α-, β-, or non-α/β-IC (1, 2). Encoded by the SLC4A1 gene, kAE1 and erythroid anion exchanger 1 (AE1) are Cl^−^/HCO_3_^−^ exchangers organized with 14 transmembrane domains (3, 4). The two proteins differ only in their N-terminal regions due to a lack of the first 65 N-terminal amino acids in the kAE1 protein (5, 6). In α-intercalated cells, V-H^+^-ATPase is located at the apical membrane whereas kAE1 is exclusively expressed at the basolateral membrane (1). A functional interplay between V-H^+^-ATPase and kAE1 contributes to the acid-base balance. Membrane permeant CO_2_ is hydrolyzed in the cytosol of α-IC by the enzymatic activity of carbonic anhydrase II (CA II) to carbonic acid (H_2_CO_3_), which spontaneously dissociates into H^+^ and HCO_3_^−^. While HCO_3_^−^ is reabsorbed into the blood at the basolateral membrane via kAE1 in a 1:1 exchange with Cl^−^ ions, secretion of H^+^ through the apical membrane is mediated by V-H^+^-ATPase. After reaction with ammonia or phosphate ions, protons are excreted as their corresponding acids in the urine (7–10).

Any malfunction of CA II, kAE1, or V-H^+^-ATPase causes distal renal tubular acidosis (dRTA), a disease in which metabolically generated protons fail to be excreted into the urine and, as a result, plasma pH becomes acidic (7). dRTA is mainly characterized by low blood pH, which indirectly leads to high urinary pH and ultimately results in nephrocalcinosis, kidney stones, metabolic acidosis, hypokalemia, and hyperchloremia as well as failure to thrive (11, 12). Two types of dRTA-causing mutations in the SLC4A1 gene exist: autosomal dominant mutations and recessive mutations (13–20). Previous studies performed in polarized Madin-Darby canine kidney (MDCK) cell lines demonstrated that disease-causing kAE1 mutations result in inactive or mislocalized anion exchangers that are either retained in the endoplasmic reticulum (ER) and/or Golgi structures or are mistrafficked to the apical membrane (8, 21, 22). Specifically, studies in MDCK cells showed that a kAE1 R589H mutation provokes a complete retention of kAE1 in the ER compartment (8, 22). In contrast, mouse inner medullary collecting duct 3 (mIMCD3) cells as well as cortical collecting duct M1 cells expressing the dominant kAE1 R589H mutation exhibited an unaltered targeting of the mutant protein to the basolateral membrane and normal ion exchange (23). Therefore, deciphering the targeting mechanism of this exchanger is necessary to clarify the controversies described in the literature.

Although significant advancement has been made in understanding the physiological and pathological roles of kAE1 in the kidney since its discovery in 1989 (24, 25), the recent *in vivo* data in mouse and from dRTA patients point to mechanisms of dRTA development that are more complex than originally assumed (23, 26). Since relatively little is known about the mechanism(s) targeting this exchanger at the basolateral membrane, it would be beneficial to better understand kAE1 transport under both normal and dRTA conditions. For this reason, in this article, we examine the potential of Saccharomyces cerevisiae as a model organism for studying specific aspects of kAE1 cell physiology. We showed that full-length kAE1 is successfully expressed in S. cerevisiae in detectable quantity after codon usage optimization. Moreover, our data confirm for the first time that full-length kAE1 variants are able to reach the yeast plasma membrane (PM) and we provide further information about intracellular kAE1 localization in yeast. Using pH measurement assays and anion-exchange chromatography, we further obtained evidence for the biological activity of kAE1. On the basis of our findings, the model organism S. cerevisiae represents a novel and suitable tool to faster address kAE1-related cell physiological questions in detail.

## RESULTS

### Codon optimization leads to heterologous expression of human kAE1 in yeast.

Previous studies already demonstrated the heterologous expression of various truncated versions of red cell anion exchanger 1 (AE1; 361 to 911 amino acids [aa], 183 to 911 aa, and 388 to 911 aa) in the bakers's yeast species S. cerevisiae (27–29). So far, the results have revealed that only AE1^361–911^ was partially transported to the PM and showed anion transport activity *in vivo*, whereas other truncated variants seemed to be trapped in intracellular membranes (28, 30). Furthermore, attempts to successfully express full-length versions of AE1 as well as kAE1 failed or resulted in inactive anion transporters unable to reach the cell surface (30).

**FIG 1:**
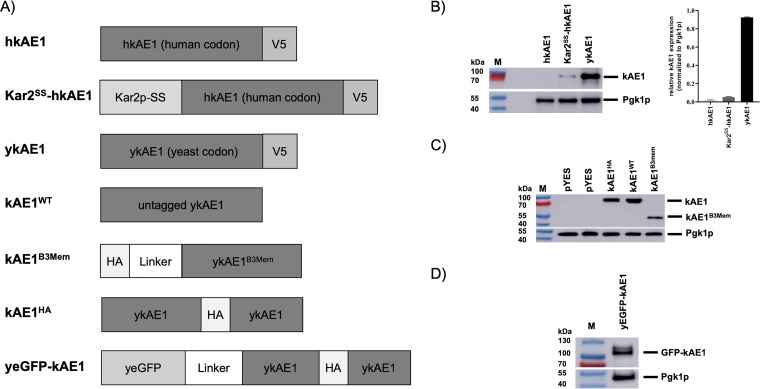
Expression of various kAE1 variants in S. cerevisiae. (A) Schematic overview of full-length and truncated kAE1 fusion proteins constructed in this study. Positions of GGGGS linker (Linker), yeGFP, and epitope tags (V5 or HA) are indicated. In the truncated kAE1 version (kAE1^B3Mem^), the first 360 amino acids of the native AE1 sequence are deleted. (B) Expression profiles (left) and quantification (right, *n* = 2) of different V5-tagged kAE1 variants containing the original human (hkAE1) or yeast codon-optimized (ykAE1) DNA sequence. The ER signal sequence of Kar2p (Kar2^SS^) was added to the N terminus of the human kAE1 sequence to improve its ER targeting (Kar2^SS^-hkAE1). A protein standard (M) was used as a size determination marker, and phosphoglycerate kinase 1 (Pgk1p) served as a loading control. (C and D) Western blot analysis of yeast cells expressing yeast codon-optimized variants of untagged wild-type kAE1 (kAE1^WT^), kAE1 containing an HA tag within the third extracellular loop between Asn^556^ and Val^557^ (kAE1^HA^), truncated kAE1 (kAE1^B3Mem^) (C), or kAE1 with an N-terminal fusion to yeast enhanced GFP (yeGFP-kAE1) (D). Pgk1p served as a loading control.

In a first step, we analyzed the potential of yeast to express full-length versions of kAE1. Therefore, three different full-length human kAE1 variants were cloned under the control of the galactose-inducible *GAL1* promoter and their expression profiles were validated in wild-type (WT) BY4742 cells via Western blot analysis (Fig. 1A). As expected and previously reported, no expression was detectable in yeast cells transformed with vectors containing the native human kAE1 sequence (Fig. 1B). Addition of an N-terminal ER signal sequence from the yeast ER chaperone Kar2p, which is frequently used to facilitate proper ER targeting of heterologously expressed proteins in yeast (31), did not dramatically improve kAE1 expression; however, a minor signal of kAE1 at ∼94 kDa was visible. Interestingly, only the modification of the whole human sequence to a yeast-optimized form of codon usage led to a remarkable level of kAE1 expression in BY4742 cells (Fig. 1B). Based on the findings indicating that human codon usage strongly limits kAE1 expression capacity in S. cerevisiae, all further constructs were adapted to the yeast-specific codon usage.

It is known that modifying the C terminus of kAE1 in mammalian cells can alter the protein's intracellular targeting (32). In contrast, alterations of the N terminus and/or integration of an epitope tag into the third extracellular loop affect neither kAE1 function nor its correct localization in mammalian cells (8, 33, 34). To exclude some negative effects of the initially tested C-terminal V5-tagged constructs, various kAE1 versions, including untagged wild-type kAE1 (kAE1^WT^), truncated kAE1 with an N-terminal hemagglutinin (HA) tag (kAE1^B3Mem^), kAE1 with an integrated HA tag within the third extracellular loop (kAE1^HA^), and kAE1 with an N-terminal fusion to yeast-enhanced green fluorescent protein (GFP) (yeGFP-kAE1), were constructed and their successful expression was confirmed by Western blotting (Fig. 1A, C, and D).

In α-intercalated cells, kAE1 is N-glycosylated at Asn^642^ (according to the AE1 amino acid sequence) during its maturation and transport to the basolateral membrane; however, mutation of Asn^642^ did not affect proper AE1 function or trafficking in eukaryotic cells (35). To check if the N-glycosylation of Asn^642^ takes place in S. cerevisiae, cell lysates from wild-type yeast expressing the different kAE1 variants were treated with endoglycosidase H (Endo H) or peptide:N-glycosidase F (PNGase F) prior to analysis by immunoblotting. In these experiments, there was no detectable size shift for any of the tested variants, indicating that full-length kAE1 versions are not glycosylated when expressed in yeast (data not shown). These findings are consistent with early reports demonstrating that AE1 is likewise not N-glycosylated when heterologously expressed in yeast (29).

In summary, these data show that full-length variants of kidney anion exchanger 1 can be successfully expressed after codon adaptation in S. cerevisiae. In contrast to results from human cells, Asn^642^ is not posttranslationally modified in yeast, leading to the presence of a nonglycosylated protein.

### Full-length kAE1 variants are partially transported to the yeast PM.

A prerequisite for using yeast as a kAE1 model system is the correct trafficking of the anion transporter to the yeast PM. Until now, detection of AE1 and/or kAE1 at the PM had been described only after truncation of its cytosolic N-terminal domain (28).

**FIG 2:**
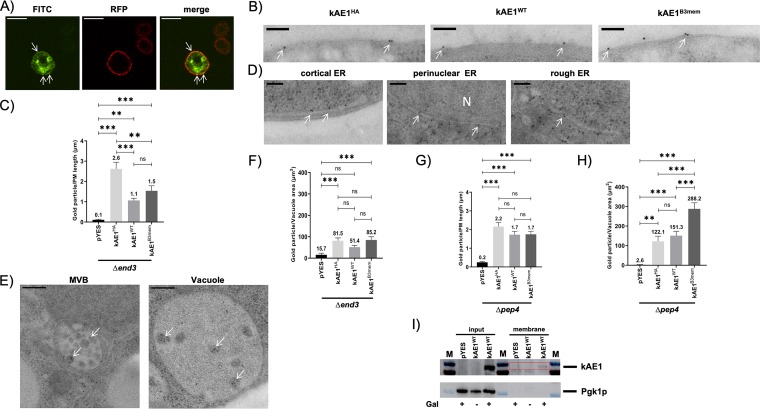
Intracellular localization of different kAE1 variants in S. cerevisiae. (A) Indirect-immunofluorescence images of kAE1^HA^ (green) and the RFP-tagged PM marker Pma1p (red) in BY4742 cells. For kAE1 detection, yeast cells were probed with primary rat anti-HA and secondary FITC-coupled anti-rat antibodies. Bar, 5 μm. (B) Transmission electron microscopy (TEM)-immunogold localization of kAE1 in Δ*end3* cells expressing yeast codon-optimized versions of untagged wild-type kAE1 (kAE1^WT^), kAE1 with an integrated HA tag within the third extracellular loop (kAE1^HA^), or truncated kAE1 (kAE1^B3Mem^) on 70-nm-thin sections. kAE1 localization was detected by the use of 10-nm-diameter colloidal gold-conjugated protein A. White arrows indicate kAE1 localized at the PM. Bar, 100 nm. (C) Statistical analysis of kAE1 immunogold particles located at the PM of Δ*end3* cells (*n* = 33 cells/sample). Mean average values ± SEM are displayed (ns, not significant; *, *P* < 0.05; **, *P* < 0.01; ***, *P* < 0.001 [one-way ANOVA]). (D and E) Subcellular localization of kAE1 in Δ*end3* cells. kAE1 immunogold labels (white arrows) were present in cortical ER, rough ER, and perinuclear ER (D) and in vacuole and multivesicular bodies (E). EM images shown are from cells expressing kAE1^HA^; however, kAE1^WT^ and kAE1^B3Mem^ also showed identical subcellular localization patterns (data not shown). Bar, 100 nm (D) or 200 nm (E). (F) Statistical evaluation of gold particles detecting immunolabel of the indicated kAE1 variants located in the vacuole of Δ*end3* cells (*n* = 26 cells/sample). Mean average values ± SEM are displayed. (G and H) Statistical analysis of kAE1 signals located in the PM (G, *n* = 33 cells/sample) or vacuole (H, *n* = 27 cells/sample) of Δ*pep4* cells. Mean average values ± SEM are displayed (ns, not significant; *, *P* < 0.05; **, *P* < 0.01; ***, *P* < 0.001 [one-way ANOVA]). (I) Cell surface biotinylation of kAE1. Wild-type cells carrying a plasmid with an untagged, yeast codon-optimized kAE1 version were cultivated in the presence (+, induced condition) or absence (-, noninduced condition) of galactose (Gal). All cultures were biotinylated by treatment with Sulfo-NHS-SS-Biotin and purified via avidin beads. Whole-cell lysates (Input) served as a control to determine the total amount of kAE1 (detected with anti-kAE1), while phosphoglycerate kinase 1 (Pgk1p) served as a cytosolic marker protein to determine the cellular integrity of the samples. The membrane fraction (surface) represents the pool of kAE1 at the cell surface (red box).

To initially determine whether kAE1 reached the PM in S. cerevisiae, kAE1^HA^ was coexpressed with the classical PM marker Pma1p (Pma1p-RFP [red fluorescent protein]) in wild-type cells and their colocalization was assessed by spinning-disc microscopy. As shown in Fig. 2A, although the green kAE1 staining partially colocalized with the red PM marker signal, the majority of kAE1 staining is visible intracellularly and does not show any colocalization with Pma1p. However, we could not exclude the possibility that the observed kAE1 staining in the cell periphery arose from cortical ER structures that were tightly associated with the yeast PM.

To further support these preliminary findings and adequately address this issue in more detail, we decided to determine the intracellular kAE1 localization by immunolabeling and subsequent transmission electron microscopy (TEM) (Fig. 2B). Therefore, two different yeast strains defective either in endocytosis (Δ*end3* mutant) or in vacuolar degradation (Δ*pep4* mutant) were transformed with galactose-inducible expression plasmids containing HA-tagged kAE1 (kAE1^HA^), wild-type kAE1 (kAE1^WT^), or a truncated anion transporter (kAE1^B3Mem^). kAE1^B3Mem^ was used as a positive control because its PM localization had already been described in yeast (28, 30), while cells containing an empty vector served as a negative control. In theory, deletion of *END3* should impair the receptor-mediated and fluid-phase endocytosis which subsequently results in an increased accumulation of kAE1 protein at the PM level (36, 37). In contrast, loss of Pep4p, a protein involved in the proper maturation of several vacuolar hydrolases, should affect only kAE1 degradation in the vacuole, allowing better visualization of kAE1 trafficking to this compartment (38, 39). Indeed, it has already been reported that the use of protease-deficient strains can significantly improve kAE1 expression levels in yeast (27, 28).

We first wanted to know if Δ*end3* cells expressing the different kAE1 variants show any kAE1 localization at the PM level. Using immunogold labeling and primary antibodies against kAE1, gold particles could be frequently found at the yeast PM in all kAE1-expressing strains (Fig. 2B). In most cases, no cortical ER structures were visible in close (∼35 nm) proximity to the labeling, indicating that these signals most likely represent kAE1 proteins located at the PM. In contrast, there was nearly no PM labeling visible in the negative-control samples (see Fig. S1 in the supplemental material). To confirm the differences in PM labeling between the samples, PM-localized gold particles were counted (*n* = 33 cells/sample) and the corresponding results are summarized in Fig. 2C. Statistical validation showed a significant increase in kAE1 localization at the PM in kAE1-expressing cells compared to the empty vector control. Although kAE1^HA^ showed the largest amount of PM signals followed by kAE1^B3Mem^ and kAE1^WT^, we could not exclude the possibility that the relative percentages of PM-localized kAE1 signals did not differ greatly between the different constructs because of their expression level variations (Fig. S2). In contrast to early reports, our electron microscopy (EM) and colocalization results nicely highlight—for the first time—that not only truncated but also full-length variants of wild-type and HA-tagged kAE1 can reach the yeast PM, at least to a minor extent.

10.1128/mSphere.00802-19.1 FIG S1 EM image of Δ*end3* cells expressing an empty vector control and probed with anti-kAE1 (BRIC170), showing some unspecific gold particles in cell wall, nucleus, and vacuole. Bar, 500 nm. Download FIG S1, JPG file, 0.4 MB. Copyright © 2020 Sarder et al. 2020 Sarder et al. This content is distributed under the terms of the Creative Commons Attribution 4.0 International license.

10.1128/mSphere.00802-19.2 FIG S2 Expression levels of the different kAE1 variants in Δ*end3* and Δ*pep4* yeast cells that were used for the electron microscopy experiments. Western blots were probed with anti-kAE1 (BRIC170) and anti-mouse HRP to visualize the expression of the indicated full-length and truncated kAE1 versions. Cells transformed with the empty vector (pYES) served as a negative control. Pgk1p was used as a loading control and was detected via anti-Pgk1 and anti-mouse HRP. Download FIG S2, JPG file, 0.3 MB. Copyright © 2020 Sarder et al. 2020 Sarder et al. This content is distributed under the terms of the Creative Commons Attribution 4.0 International license.

Additionally, immunogold labeling in all three kAE1 constructs likewise showed that kAE1 is localized in structures belonging to cortical ER, rough ER, perinuclear ER, and vacuole (Fig. 2D and E). In contrast, control samples containing the empty vector showed only a minimal amount of unspecific labeling at the cell wall, vacuole, and nucleus resulting from unspecific binding of the used secondary antibody and/or linker fragment (Fig. S3). Moreover, kAE1 signals were also associated with multivesicular bodies (MVBs), indicating that a portion of the anion transporter is presumably degraded via the yeast vacuole (Fig. 2E). In yeast, proteins from the PM or *trans-*Golgi network can be generally sorted in MVBs and delivered to the vacuole via endosomal sorting complexes required for transport (ESCRT) pathways (40). Since MVB-associated kAE1 signals were observed in Δ*end3* cells, which are defective in receptor-mediated endocytosis, our results indicate that kAE1-containing MVBs most likely originated from the *trans*-Golgi network. However, it was shown previously that an *END3* deletion strongly impairs but does not completely disturb internalization of PM proteins such as α-factor receptor Ste3p. Therefore, a definitive conclusion concerning whether the maturation of kAE1-containing MVBs starts from the PM or the *trans*-Golgi network cannot be drawn (37). To better assess whether kAE1 is truly delivered to the vacuole in Δ*end3* cells, the number of gold particles present in the vacuole in each sample after kAE1 immunolabeling was determined. As shown in Fig. 2F, kAE1-expressing cells showed a significant increase of gold particles in the vacuole compared to the control samples.

10.1128/mSphere.00802-19.3 FIG S3 EM image of Δ*end3* cells expressing an empty vector and probed only with a secondary antibody and linker fragment, illustrating some unspecific gold particles in the cell wall and the nucleus. A similar labeling pattern was seen in empty vector control cells probed with both primary and secondary antibodies (compare with Fig. S1). Bar, 500 nm. Download FIG S3, JPG file, 0.5 MB. Copyright © 2020 Sarder et al. 2020 Sarder et al. This content is distributed under the terms of the Creative Commons Attribution 4.0 International license.

A similar intracellular localization of kAE1 was detected also in the *Δpep4* deletion background, and the results are summarized in Fig. S4. Numbers of gold particles at PM-localized kAE1 were similar to the number seen with the Δ*end3* mutant (Fig. 2G). Notably, all constructs showed a significantly increased accumulation of kAE1 signals in the vacuole after deletion of the major protease Pep4p (Fig. 2H). It seems as if a significant fraction of kAE1 is delivered to the vacuole for Pep4p-dependent degradation.

10.1128/mSphere.00802-19.4 FIG S4 Subcellular localization of kAE1 in Δ*pep4* cells. kAE1 signals (black arrows) are detectable in structures belonging to the plasma membrane, cortical ER, rough ER, and perinuclear ER. Bar, 100 nm. EM images of the vacuole are from cells expressing kAE1^B3Mem^, whereas the other sections derived from cells expressing kAE1^HA^. Bar, 200 nm. Download FIG S4, JPG file, 1.0 MB. Copyright © 2020 Sarder et al. 2020 Sarder et al. This content is distributed under the terms of the Creative Commons Attribution 4.0 International license.

To further support and biochemically address the observation of PM localization of kAE1, cell surface biotinylation was performed on wild-type yeast cells expressing untagged wild-type kAE1 followed by an avidin pulldown to detect the pool of PM-localized kAE1. Although biotinylation of PM proteins in yeast is hard to establish due to the cell wall barrier and the strong reduction of the biotinylation efficiency by additional labeling of cell wall proteins (41–43), we succeeded in detecting minor amounts of kAE1 in the biotinylated cell surface fraction, while no signals were visible either under noninduced conditions or in cells expressing an empty control vector (Fig. 2I).

### Kidney AE1 mainly accumulates in membrane-like structures derived from the ER.

**FIG 3:**
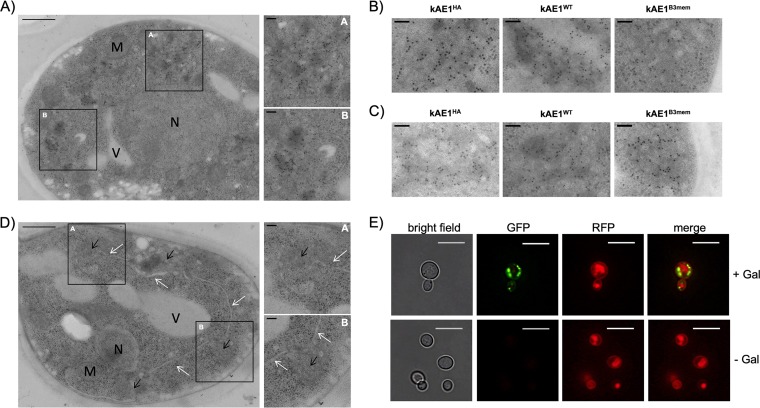
kAE1 accumulates in intracellular membrane/vesicle-like structures. (A) TEM-immunogold labeling of kAE1 protein. The TEM images show typical MV-like structures induced by kAE1^HA^ expression in Δ*end3* cells. Bar, 500 nm. Insets A and B show a magnified area of the marked regions (black box). Bar, 100 nm. M, membrane; N, nucleus; V, vacuole. (B and C) Accumulation of various kAE1 fusion proteins in Δ*end3* (B) and Δ*pep4* (C) cells. Bar, 100 nm. (D) MV-like structures (black arrows) are directly associated with typical ER structures (white arrows). Bar, 500 nm. Insets A and B show magnified areas of the marked regions (black box). Bar, 100 nm. (E) Fluorescence microscopy images of wild-type cells expressing N-terminal GFP-tagged kAE1 together with RFP-tagged Pma1p. GFP signals mainly localized in intracellular structures and did not colocalize with the red Pma1p signals located in the PM and vacuole (note that internalized Pma1p-RFP is transported to the vacuole, which allows vacuole staining). Bar, 10 μm.

Although a minor pool of kAE1 enters the secretory pathway and is subsequently targeted to the PM, this transport seems to be very inefficient in S. cerevisiae. It is obvious that the majority of kAE1 proteins accumulate and/or form aggregates in intracellular membrane/vesicle-like (MV) structures (Fig. 3A; see also Fig. S5). Interestingly, these structures were detectable only after kAE1 expression and were not seen in control samples. Furthermore, whether full-length or truncated versions were expressed in Δ*end3* or Δ*pep4* cells made no difference; all strains showed this feature (Fig. 3B and C). In some sections throughout the cell, a connection between the MV-like structures and the ER was obvious (Fig. 3D), indicating that the MV structures derived from the ER. However, the accumulated kAE1 signals might also be associated with the Golgi apparatus or with endosomes, which is impossible to judge without any marker proteins in yeast.

10.1128/mSphere.00802-19.5 FIG S5 Detailed EM image of membrane/vesicle-like structures in Δ*end3* cells expressing kAE1^HA^. Gold-labeled kAE1 signals are visible in membrane structures and vesicles. Bar, 100 nm. Download FIG S5, JPG file, 0.4 MB. Copyright © 2020 Sarder et al. 2020 Sarder et al. This content is distributed under the terms of the Creative Commons Attribution 4.0 International license.

Because intracellular kAE1 accumulation was also detected in fluorescence images after yeGFP-kAE1 (Fig. 3E) or kAE1^HA^ (Fig. 2A) expression, further colocalization studies performed with specific fluorescence-tagged organelle markers (e.g., for the Golgi network, ER, and endosomes) would represent an elegant way to determine the precise localization of the observed kAE1 aggregates. So far, most of the signals were found to be clearly distinguishable from the PM and/or vacuole structures, which were both stained by the RFP-tagged PM marker Pma1p (Fig. 3E). Since those findings were not within the scope of our present study, we did not perform further experiments in that direction. However, it will be important to address this phenomenon in more detail in the near future and to identify conditions which can prevent kAE1 accumulation in intracellular organelles.

### Human kAE1 shows biological activity in yeast.

**FIG 4:**
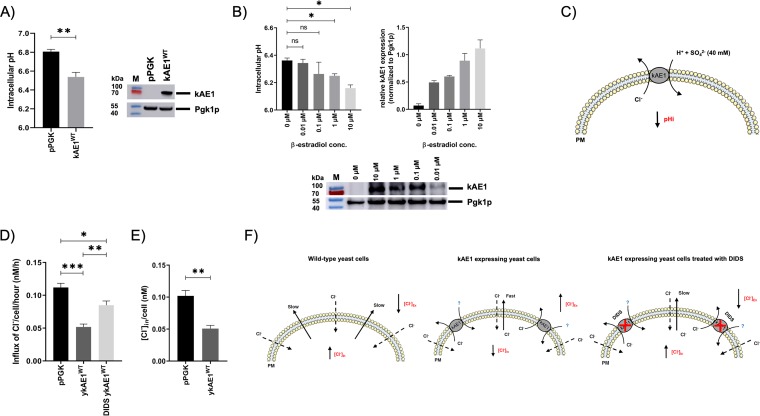
kAE1 expression decreases intracellular pH and affects intracellular chloride concentration. (A) pH measurements of wild-type BY4742 cells expressing pPGK (negative control) or pPGK-kAE1^WT^ (*n* = 4). Cytosolic pH data are displayed as mean average values ± SEM (ns, not significant; *, *P* < 0.05; **, *P* < 0.01; ***, *P* < 0.001 [one-way ANOVA]). Western blot analysis was performed as an expression control, and blots were probed with anti-kAE1 (BRIC170) and anti-mouse antibodies coupled with horseradish peroxidase (HRP). (B) pH measurement of β-estradiol-inducible BY4742-GEV cells expressing pYES-kAE1^WT^ with the indicated β-estradiol concentration (*n* = 4). Cytosolic pH data are displayed as mean average values ± SEM (ns, not significant; *, *P* < 0.05; **, *P* < 0.01; ***, *P* < 0.001 [one-way ANOVA]). Western blot analysis was performed to quantify kAE1 expression; blots were probed with anti-kAE1 and anti-rabbit antibodies coupled with HRP. Relative kAE1 expression levels were normalized to Pgk1p and then calculated from Western blot analyses (*n* = 2). (C) Hypothetical model explaining the observed pH drop in the yeast cytosol after kAE1 expression. After induction of protein expression, a minor amount of kAE1 is transported to the yeast PM, where it can fulfill its biological function. Due to the absence of HCO_3_^−^ in the experimental setup, kAE1 presumably exports cytosolic Cl^−^ in exchange with SO_4_^2-^ + H^+^ (medium contains 40 mM SO_4_^2-^). It was previously reported that kAE1 can likewise promote the influx or efflux of SO_4_^2-^ coupled with H^+^ in yeast cells (29, 60). The additional influx of H^+^ subsequently decreases the cytosolic pH in yeast (pHi). (D) Anion exchange chromatography of wild-type BY4742 cells containing pPGK or pPGK-kAE1^WT^ in the presence or absence of 200 μM DIDS. The data representing the Cl^−^ influx rate per hour per cell (indicated in nanomoles; *n* = 4) are illustrated as means ± SEM (*, *P* < 0.05; **, *P* < 0.01; ***, *P* < 0.001 [one-way ANOVA]). The Cl^−^ influx rate was normalized to total cell number (OD_600_ = 1, corresponding to 1 × 10^7^ cells). (E) Intracellular chloride concentrations ([Cl^−^]_in_; indicated in nanomoles) of cells expressing empty vector or pPGK-kAE1^WT^ (*n* = 3). Data are normalized to total cell numbers and illustrated as means ± SEM (ns, not significant; *, *P* < 0.05; **, *P* < 0.01; ***, *P* < 0.001 [one-way ANOVA]). (F) Hypothetical model explaining the observed reduction in kAE1-mediated influx of Cl^−^ in the presence and absence of the kAE1 inhibitor DIDS. Under hyperosmotic conditions (150 mM NaCl), Cl^−^ passively (dashed arrow) diffuses into the cytosol, subsequently increasing the intracellular Cl^−^ concentration ([Cl^−^]_in_) of wild-type yeast cells and leading to a drop in the level of extracellular Cl^−^ ([Cl^−^]_ex_). In the presence of kAE1, intracellular Cl^−^ seems to be excreted (solid arrow) as a consequence of the action of kAE1, leading to a decrease in [Cl^−^]_in_ and an increase in [Cl^−^]_ex._ So far, it has been unclear which anion is cotransported with Cl^−^ (illustrated as a question mark). In contrast to the pH measurements, there is no excess of SO_4_^2-^ in the medium which would explain the observed efflux of Cl^−^ in exchange with SO_4_^2-^. However, DIDS treatment, which selectively inhibits kAE1 function, causes a wild-type-like phenotype with higher [Cl^−^]_in_ and lower [Cl^−^]_ex_ levels.

kAE1-mediated influx/efflux of Cl^−^ is known to affect the intracellular pH of eukaryotic cells (44, 45). Since the expression of wild-type kAE1 is associated with a significant drop in the steady-state pH level in mIMCD3 cells (7), we asked whether constitutive kAE1 expression likewise modulates the intracellular steady-state pH in yeast cells. For measuring the intracellular pH, yeast cells constitutively expressing wild-type kAE1 (pPGK-kAE1^WT^) or empty vector (pPGK) were incubated with the ratiometric pH indicator SNARF-5F-AM and the intracellular steady-state pH was measured as described in Materials and Methods. pH-sensitive and cell-permeative SNARF 5F dyes have been used previously to determine the cytosolic pH in yeast cells (46–48). As illustrated in Fig. 4A (see also Fig. S6), kAE1 expression significantly reduced the cytosolic pH in wild-type cells. To further link these initial findings to the presence of the anion transporter, cytosolic pH was also calculated in cells with a stepwise induction of kAE1 expression using a genetically modified BY4742 wild-type strain containing a β-estradiol-inducible Gal4dbd.ER.VP16 (GEV) promoter system (49). In agreement with the findings described above, we observed that higher kAE1 expression levels resulted in a gradual and dose-dependent reduction in the cytosolic pH (Fig. 4B).

10.1128/mSphere.00802-19.6 FIG S6 pH calibration curves from BY4742 cells expressing empty vector (left) or kAE1^WT^ (right) that had been used for the pH measurements whose results are shown in Fig. 4A. Mean values ± SEM are indicated (*n* = 2). Download FIG S6, JPG file, 0.1 MB. Copyright © 2020 Sarder et al. 2020 Sarder et al. This content is distributed under the terms of the Creative Commons Attribution 4.0 International license.

Finally, we tested whether kAE1 expression is capable of altering Cl^−^ influx in yeast. Changes in extracellular and intracellular Cl^−^ concentrations in wild-type cells expressing pPGK or pPGK-kAE1^WT^ were measured via anion-exchange chromatography (Fig. 4D and E). Surprisingly, and in contrast to previous ^36^Cl^−^ uptake studies (28), decreased intracellular Cl^−^ concentrations as well as diminished levels of Cl^−^ influx were simultaneously detected in the presence of kAE1, indicating that an efflux of Cl^−^ instead of an influx occurred under these conditions. In contrast, cells expressing an empty vector showed an increased level of Cl^−^ influx and thus, an elevated intracellular Cl^−^ concentration compared to kAE1-expressing cells (Fig. 4D and E). As expected, inhibition of kAE1 function by treatment with the kAE1 inhibitor DIDS (disodium 4,4′-diisothiocyanatostilbene-2,2′-disulfonate) prevented the kAE1-mediated Cl^−^ efflux, leading to an increased level of Cl^−^ influx compared to kAE1-expressing cells. Although elevated Cl^−^ influx after kAE1 expression was expected and was already described for the truncated AE1^361–911^ variant (28), the significant differences between empty vector and wild-type kAE1-expressing cells are pointing toward biological activity of the heterologously expressed anion transporter in yeast. Hypothetical models drawn from the obtained results are depicted in Fig. 4C and F.

## DISCUSSION

Yeast has been widely used to study not only expression, localization, and functionality but also structure and interaction partners of many renal proteins (50). However, it is known that human membrane proteins, when expressed in yeast, show less maturation than the corresponding fungal or plant homologs and often fail to reach their correct intracellular localization and function (16). Nevertheless, previous studies already demonstrated that yeast is suitable for high-level expression of different AE1 variants (27, 29, 30). However, efforts to express full-length AE1/kAE1 variants either failed or resulted in inactive transporter variants which were incapable of reaching the yeast PM (30). Since only truncated AE1 variants show biological activity, S. cerevisiae was primarily used to study structural aspects or as an interaction partner with these variants (7, 27, 29, 30, 51, 52).

Our presented results demonstrate that human codon usage was the limiting factor for full-length kAE1 expression in yeast, a very common issue in heterologous protein expression in a foreign organism (53, 54). On the basis of our data, it is now possible to express a wide range of tagged and untagged full-length kAE1 variants in detectable quantities. However, modification of the N terminus of kAE1 most likely negatively affects its proper import into the yeast ER, as shown here by the strongly reduced expression level of eGFP-kAE1.

In contrast to earlier reports (30), our electron microscopy data strongly suggest localization of at least a minor fraction of full-length kAE1 protein at the yeast PM as visualized for wild-type, HA-tagged, and truncated kAE1. Furthermore, a previously proposed hypothesis postulating that the N-terminal part from amino acid 182 to amino acid 360 of kAE1 prevents its correct targeting to the yeast PM seems to be unlikely, because we did not observe a significant difference in PM localization between truncated and wild-type kAE1 (27, 30). Although the analysis clearly showed a cell surface localization of all kAE1 variants, it is not possible to quantify the percentage of PM-localized kAE1 in the different constructs. Dissimilar labeling efficiencies of the cellular substructures in EM studies prevent quantification and comparison of two or more cellular structures at the same time. Compared to the already reported PM localization of kAE1^B3mem^, full-length versions are transported to the cell surface in at least equal numbers.

Furthermore, we were able to assess the intracellular localization of full-length and truncated kAE1 variants; in that assessment, all variants showed very similar intracellular localization patterns. The presence of kAE1 in MVBs indicates that kAE1 is partially transported to and degraded in the yeast vacuole. Higher-density localization of kAE1 in vacuoles in the Δ*pep4* background of kAE1^WT^- and kAE1^B3mem^-expressing cells further supports this assumption. Since endocytosis-defective Δ*end3* cells showed significant more kAE1 immunogold label in the vacuole than the negative control, ESCRT-mediated transportation from the Golgi network to the vacuole is more likely than the alternative route from the PM to the vacuole. Additional spinning-disc microscopy and cell surface biotinylation data further support the EM results and once again show that a minor amount of full-length kAE1 is localized at the PM.

Previous studies in yeast already postulated a strong tendency of AE1 to be trapped in intracellular membranes (29). In the EM pictures, it was obvious that the vast majority of kAE1 was intracellularly accumulating in protein-rich membrane/vesicle-like structures (visible as dense structures in the EM pictures). The aggregate formation was specifically induced by kAE1 expression, regardless of which kind of kAE1 construct was expressed. Interestingly, the close proximity of these structures to the ER suggests the conclusion that most kAE1 molecules are presumably trapped in the ER or in ER-derived vesicles and do not efficiently enter the secretory pathway. In the literature, there are many examples of an accumulation of heterologously expressed proteins in intracellular ER structures in yeast (55, 56). So far, our data have not adequately addressed the reason for this massive accumulation. It is conceivable that the protein overexpression induces folding defects which subsequently prevent kAE1 from further trafficking. Moreover, the observed lack of Asn^642^ glycosylation in full-length kAE1 might prevent its efficient entry into the secretory pathway; although normal trafficking of an unglycosylated kAE1 to the PM was observed previously in human cells (35, 57). Eventually, structural characteristics influence correct insertion and/or folding of kAE1 in yeast. Previous yeast studies demonstrated that overexpression of glycophorin A significantly increases the PM targeting of truncated kAE1^B3Mem^ (30). Since our EM pictures of kAE1^B3Mem^ also show the accumulation phenotype, it is likewise possible that a specific interaction partner—responsible for efficient transport of kAE1 to the PM—is simply missing in the model organism. For instance, it would be interesting to coexpress other known AE1/kAE1 interaction partners such as glycophorin A or the integrin-linked kinase (ILK) and determine their effect on the amount of PM-localized kAE1 (58, 59). However, additional experiments are needed to adequately answer these issues and to presumably optimize the efficiency of kAE1 transport to the cell surface.

Our pH measurement results indicate the functionality of full-length kAE1 in yeast. The occurrence of a drop in the cytosolic pH level in a kAE1 dose-dependent manner is in line with recent data from Lashhab and coworkers, who showed that overexpression of wild-type kAE1 reduces the steady-state pH level in mammalian cells (7). In addition, it is further known that AE1/kAE1 is also capable of transporting sulfate (SO_4_^2-^) instead of bicarbonate (HCO_3_^−^) in exchange with Cl^−^ in both directions through the yeast PM (29, 60, 61). To perform this exchange step, coupled transportation of SO_4_^2-^ and H^+^ is required (60). As illustrated in our model, the extracellular excess of SO_4_^2-^ and the absence of HCO_3_^−^ in our experimental setup could presumably lead to a proton-coupled influx of SO_4_^2-^ and an efflux of Cl^−^, decreasing the intracellular pH observed in our experiments. In line with this theory, the stepwise increase of kAE1 expression caused a gradual pH decrease, most likely induced by the presence of an increasing amount of active kAE1 protein at the cell surface under conditions of higher expression levels.

By using anion-exchange chromatography, we also observed significant differences between the chloride influx and the intracellular chloride concentration in kAE1- and empty vector-expressing cells, pointing toward a biological activity of kAE1. However, it is difficult to explain why the presence of kAE1 results in an efflux of Cl^−^, thus lowering the intracellular Cl^−^ level. Under hyperosmotic conditions (150 mM NaCl), it has been shown that passive Cl^−^ diffusion into the cytosol takes place (62). In our opinion, it is possible that passively diffused Cl^−^ is continuously exported by the action of PM-localized kAE1. In contrast, cells expressing the empty vector are not able to perform such Cl^−^ efflux in a similar way. As expected, inhibition of kAE1 with DIDS diminished the observed efflux of chloride in kAE1-expressing cells, indicating that the kAE1 exchange function is required for the reduced Cl^−^ influx in yeast. Nevertheless, the results are in contrast to previous studies in yeast showing an influx of Cl^−^ in cells expressing a truncated AE1 variant (28). Regrettably, we were unable to compare the anion exchange data with data from uptake studies using radioactively labeled ^36^Cl^−^ due to the completely different experimental conditions. Furthermore, relatively little is known about the underlying mechanisms regulating pH and Cl^−^ transport in yeast, which makes it difficult to fully explain the obtained data. However, both experimental setups suggest biological functionality of full-length kAE1, but further studies will be required to better understand the underlying processes in yeast.

In sum, our data highlight the potential of the yeast S. cerevisiae to serve as a model organism for the analysis of nontruncated variants of human kidney anion exchanger 1. On the basis of the presented data, yeast represents a model “prototype” combining many advantages over higher eukaryotic systems such as fast cell growth, inexpensive maintenance, and amenability to a large number of genetic manipulation tools. As with every model system, S. cerevisiae also displays some limitations for studying (kAE1) physiology/pathology. Indeed, yeast represents a single-cell organism which is not polarized, excluding the possibility of studying the role of cell-to-cell interactions as they occur in a complex organ such as the kidney (15). Likewise, it is not possible to study the complex dRTA-causing context. However, the presence of kAE1 at the cell surface—albeit to a small extent—demonstrates that the anion transporter is generally capable of reaching its final PM destination in yeast, providing a good starting point for future optimization. A larger amount of PM-localized kAE1 would presumably help us to better detect kAE1 activity and to dissect the mechanism(s) of kAE1 trafficking. At the moment, the system is limited to single-gene overexpression screens (e.g., open reading frame [ORF] libraries) to identify candidates for improving intracellular kAE1 transportation to the PM. In addition, the results from our initial pH measurements and anion-exchange chromatography analyses provide a first body of evidence indicating a biological activity of full-length kAE1—albeit that activity is not yet mechanistically understood—and now allow rapid screening of the complete kAE1 sequence (including the N-terminal part) for mutations affecting transport activity. In future experiments, we will address the intracellular accumulation issue in more detail in order to hopefully obtain an optimized yeast system which will allow us to perform a more detailed analysis of kAE1 transport to the cell periphery.

## MATERIALS AND METHODS

### Cultivation and transformation of yeast cells.

S. cerevisiae strains used in this study and listed in Table S1 in the supplemental material were routinely grown at 30°C in standard yeast extract-peptone-dextrose (YPD) complex, synthetic complete (SC), or dropout (d/o) media containing 2% glucose or 3% galactose. Yeast transformation was performed as previously described (43).

10.1128/mSphere.00802-19.7 TABLE S1 Yeast strains used in this study. Download Table S1, PDF file, 0.1 MB. Copyright © 2020 Sarder et al. 2020 Sarder et al. This content is distributed under the terms of the Creative Commons Attribution 4.0 International license.

### Vector construction.

For construction of the expression plasmids pYES-hkAE1, pYES-Kar2^SS^-hkAE1, and pYES-ykAE1, a synthetic cDNA sequence of human or yeast codon-optimized kAE1 (GeneArts, Thermo Scientific) (see Data Set S1A to C in the supplemental material) was integrated into pYES2.1 (Thermo Scientific) via TOPO cloning. The pYES2.1 vector contains a galactose-inducible *GAL1* promoter, and expression of the kAE1 variants was induced by culturing the cells in uracil d/o medium containing 3% galactose as the carbon source. Based on the expression results, a yeast codon-optimized cDNA sequence of kAE1 was used for all additional plasmids. Thereby, pYES-kAE1^HA^ and pYES-kAE1^B3mem^ were generated in the same way as described above with the exception of the cDNA sequence used (Data Set S1D and E).

10.1128/mSphere.00802-19.10 DATA SET S1 DNA sequences (5′ to 3′ direction) ordered for the construction of the different kAE1 expression plasmids. Restriction sites are illustrated in lowercase letters, epitope tag sequences are indicated in bold, and linker sequences are underlined. (A) V5-tagged human kAE1 sequence. (B) V5-tagged human kAE1 sequence with an additional Kar2p signal sequence at the N terminus. (C) V5-tagged yeast codon-optimized kAE1 sequence. (D) Yeast codon-optimized kAE1 sequence with an integrated HA tag in the third extracellular loop between aa 557 and 578 (according to the AE1 sequence). (E) Sequence of an N-terminal HA-tagged, truncated kAE1 version (aa 361 to 911). Download Data Set S1, PDF file, 0.1 MB. Copyright © 2020 Sarder et al. 2020 Sarder et al. This content is distributed under the terms of the Creative Commons Attribution 4.0 International license.

Unmodified wild-type kAE1 (kAE1^WT^) was generated by PCR with primers listed in Table S2. Finally, yeast cells were cotransformed with the corresponding PCR product and the linearized pYES2.1 vector (digested with XhoI*/*BamHI), and subsequent pYES-kAE1^WT^ plasmid ligation was performed by the use of the yeast itself via *in vivo* recombination (63). For pYES-yeGFP-kAE1, yeGFP was initially amplified by PCR with the appropriate primers using pYES2.1-yeGFP as the template (Table S2). Finally, the PCR product and linearized pYES-kAE1^HA^ vector (digested with XhoI) were transformed into yeast and ligated via *in vivo* recombination.

10.1128/mSphere.00802-19.8 TABLE S2 Primers used in this study (restriction sites are shown in lowercase letters, and linker sequences are underlined). Download Table S2, PDF file, 0.05 MB. Copyright © 2020 Sarder et al. 2020 Sarder et al. This content is distributed under the terms of the Creative Commons Attribution 4.0 International license.

For constitutive expression of kAE1, the unmodified wild-type kAE1 sequence (kAE1^WT^; see above) was digested with XhoI/BamHI and ligated into the pPGK vector to obtain pPGK-kAE1^WT^. All DNA sequences generated by PCR were verified by commercial sequencing (GATC).

### Electron microscopy and immunolocalization.

Log-phase yeast cultures (optical density at 600 nm [OD_600_] of 0.6 to 0.8) of Δ*end3* or Δ*pep4* strains expressing the indicated kAE1 variant or an empty vector control were filtered into a paste, which was pipetted into a 0.2-mm-deep aluminum carrier (Engineering Office, M. Wohlwend GmbH, Sennwald, Switzerland) and cryoimmobilized by high-pressure freezing using hpm010 (Abra fluid, Widnau, Switzerland). Freeze substitution of the cells was done using an EM-AFS2 freeze substitution device (Leica Microsystems, Vienna, Austria). The freeze substitution solution used contained 0.1% (wt/vol) uranyl acetate dissolved in anhydrous acetone, and the samples were substituted at –90°C for 24 h. The temperature was subsequently increased to –45°C at a rate of 5°C/h followed by 5 h of incubation at –45°C. The samples were rinsed three times with acetone for 10 min followed by infiltration using Lowicryl HM20 (Polysciences, Warrington, PA, USA) at –25°C with 25% Lowicryl in acetone 2 h, 50% Lowicryl for 2 h, and 75% Lowicryl for 2 h. Samples were finally maintained in 100% Lowicryl three times (10 h each) before the onset of polymerization. UV polymerization was applied for 48 h at –25°C and the temperature was increased to 20°C at a rate of 5°C per hour. The samples were left exposed to UV at room temperature (RT) for 48 h.

Thin (70-nm) sections were cut with a Leica UC6 microtome (Leica Microsystems, Vienna, Austria) and collected on Formvar-coated copper slot grids. Immunogold labeling was done by floating grids on drops of blocking buffer consisting of 1.5% bovine serum albumin (BSA) and 0.1% fish skin gelatin (in phosphate-buffered saline [PBS]) for 30 min. Incubation on drops with commercially available anti-kAE1 antibodies (BRIC170, International Blood Group Reference Library [IBGRL], Bristol, United Kingdom) derived from mouse (diluted 1:100 in blocking buffer) was conducted for 30 min, followed by a 20-min incubation with the appropriate secondary antibody (anti-mouse, Dako, catalog no. Z025902-2). Subsequently, gold-conjugated protein A with a particle size of 10 nm (Center Utrecht, Utrecht, Netherlands) was added for 20 min. The grids (floating on 5 drops of PBS) were rinsed after each incubation step. The sections were then fixed in 1% glutaraldehyde–PBS for 5 min before they were rinsed in 5 drops of water and subjected to poststaining using uranyl acetate and lead citrate.

The sections were viewed using a JEOL JEM-1400 electron microscope (JEOL, Tokyo) operating at 80 kV and equipped with a 4K TemCam F416 camera (Tietz Video and Image Processing Systems GmBH, Gautig, Germany).

Labeling at the PM was examined, and gold particles within a 32-nm range were scored for PM-localized kAE1 proteins in the different yeast strains. To calculate the number of gold particles per square micrometer of PM length, a grid consisting of 0.2-μm^2^ sections was put on each picture via the use of the ImageJ plugin Grid and the number of intersections with the PM was counted using the ImageJ plugin cell counter. Then, all data were integrated in the following equation: number of gold particles per micrometer of PM length = number of PM-localized gold particles counted/number of intersections × intersection length × π/4.

For analysis of gold particles located in the yeast vacuole, all gold particles inside the vacuole were first counted and scored for vacuole-located kAE1 proteins in the different yeast strains. Sections without any vacuoles were excluded from the analysis. To calculate the number of gold particles per square micrometer (μm^2^) of vacuole, a grid pattern (0.1-μm^2^ sections) was placed on the picture, and all corners of the grid pattern that fully located inside the vacuole were counted. Finally, all data were integrated into the following equation: number of gold particles per square micrometer of vacuole = number of vacuole-localized gold particles counted/number of corners × (intersection length)^2^.

### Cell surface biotinylation.

Yeast cells expressing wild-type kAE1 were grown to the exponential phase (OD_600_ to 1 to 1.5), harvested, and subsequently used for cell surface biotinylation. The experimental setup was as reported previously (43). In brief, yeast cells were washed with cold PBS (pH 7.2) and labeled for 90 min with Sulfo-NHS-SS-Biotin (1 mg/ml) in PBS at 4°C. After quenching of the biotinylation reaction, cells were lysed and their lysates were immediately used for pulldown with avidin agarose beads (Pierce) followed by several washing steps that included the use of wash buffer and SWS buffer (0.1% Triton X-100–PBS [pH 7.4], 350 mM NaCl, 5 mM EDTA). Bound proteins (membrane fraction) were eluted in 3× SDS buffer containing 50 mM dithiothreitol (DTT) and 5% 2-mercaptoethanol, and aliquots of lysate (input) and the membrane fraction were used for SDS-PAGE and Western blot analysis. By using antibodies against intracellular phosphoglycerate kinase 1 (Pgk1p), cellular integrity was checked during the labeling step. Anti-kAE1 antibody was used to detect the successful labeling of kAE1.

### Western blot analysis.

SDS-PAGE was performed under nonreducing conditions in 10% Tris-Tricine gels using a buffer system according to the method described in reference 64. Semidry blotting onto polyvinylidene difluoride (PVDF) membranes was carried out in transfer buffer (25 mM Tris, 190 mM glycine, 0.1% SDS, 20% methanol). In general, expression of the kAE1 constructs was validated by using primary antibodies against the N-terminal region of kAE1 (BRIC170, recognizing an epitope in the region of amino acid 368 to amino acid 382, or anti-kAE1 [52], recognizing the N terminus of kAE1 in the region of amino acid 60 to amino acid 85) and visualized with secondary horseradish peroxidase (HRP)-conjugated anti-rabbit or anti-mouse IgG antibodies. Detection of V5-tagged kAE1 variants was performed by using anti-V5 and anti-mouse HRP antibodies. For N-terminal yeGFP tagged kAE1, blots were probed with anti-GFP and anti-mouse HRP antibodies. Pgk1p (phosphoglycerate kinase 1) served as a loading control, and blots were incubated with primary anti-Pgk1 and HRP-coupled anti-mouse antibodies for detection. After incubation with SuperSignal West Femto maximum sensitivity substrate (Thermo Scientific), signals were visualized with an Amersham Imager 600 instrument (GE Healthcare). Antibody dilutions and sources are described in Table S3.

10.1128/mSphere.00802-19.9 TABLE S3 Antibodies used in this study. Download Table S3, PDF file, 0.1 MB. Copyright © 2020 Sarder et al. 2020 Sarder et al. This content is distributed under the terms of the Creative Commons Attribution 4.0 International license.

### Fluorescence microscopy.

To prevent cell movement, 20-μl volumes of living yeast cells were spotted on poly-l-lysine-coated coverslips and preincubated for 15 min. Dual-color images of yeGFP and monomeric RFP (mRFP) fusion proteins were obtained via fluorescence microscopy using a Keyence BZ-8000 microscope (100× oil immersion Plan Apo VC objective [1.4 numerical aperture {NA}]) with the preinstalled filter sets as well as standard settings for detection of GFP (488 nm) and mRFP (584 nm).

### Spinning-disc microscopy.

For indirect-immunofluorescence assays, yeast cells were first cultivated to reach an OD_600_ of 1. Next, cells (final OD_600_ of 10) were harvested by centrifugation (8,000 rpm, 5 min, RT), washed two times with 0.1 M PBS, and fixed with 3.7% formaldehyde at 20°C on a roller drum for 1 h. After three washing steps were performed with 0.1 M PBS, cells were washed two times with 1.2 M sorbitol, resuspended in 1 ml 1.2 M sorbitol supplemented with 10 μl β-mercaptoethanol and 20 μl zymolyase (5 mg/ml zymolyase 100T), and incubated for 45 min at 30°C. Finally, cells were centrifuged at 2,000 rpm for 5 min, washed two times with 1.2 M sorbitol, and used for antibody staining.

Prior antibody staining, cells were permeabilized with 0.1% SDS for 10 min at RT. Then, 30-μl volumes of the sample were spotted on poly-l-lysine coated coverslips, preincubated for 20 min, washed once with 1% BSA (in a solvent with 0.1 M PBS), and blocked for 30 min in 1% BSA. For detecting kAE1^HA^, cells were incubated at 20°C with primary anti-HA antibodies (1 h, 1:40 diluted) and secondary anti-rat antibodies conjugated with fluorescein isothiocyanate (FITC) (1 h, 1:160 diluted). After three washing steps with 1% BSA, cells were analyzed via spinning-disc microscopy.

Confocal images were acquired using an inverted microscope (Ti-Eclipse; Nikon) equipped with a Yokogawa spinning-disk unit (CSU-W1; Andor Technology). Images were acquired with a 100× oil Plan Apo total internal reflection fluorescence (TIRF) objective (Nikon) and recorded on a digital scientific complementary metal oxide semiconductor (sCMOS) camera (Orca-Flash 4.0; Hamamatsu). Image analysis was performed with Fiji software. For figure processing, the channels of each image were separated, and the levels of signal in all channels were separately adjusted to facilitate the observation of dim structures.

### pH measurement.

Intracellular pH (pHi) measurements were performed with the pH-sensitive probe SNARF-5F AM [SNARF-5F 5 (and 6)-carboxylic acid, acetoxymethyl (AM) ester, acetate; Molecular Probes]. In brief, 50-ml volumes of yeast cells transformed with empty vector pPGK (negative control) or pPGK-kAE1^WT^ were grown overnight in SC medium (pH = 6.4, 50 mM NaCl, sterilized) to the stationary phase (OD_600_ of 3 to 4) and aliquots of cells corresponding to an OD_600_ of 20 were subsequently harvested at 8,000 rpm for 5 min. After washing of the cells in 0.1 M potassium phosphate buffer (pH = 6.4 [pH was adjusted by mixing 72.2 ml of 1.0 M KH_2_PO_4_ and 27.8 ml of 1.0 M K_2_HPO_4_ with 900 ml distilled H_2_O]), cells were incubated in 0.1 M potassium phosphate buffer containing 10 μM SNARF-5F AM for 1 h at 30°C and 220 rpm. To get rid of excess amounts of SNARF-5F AM, cells were washed three times with 0.1 M potassium phosphate buffer, harvested at RT for 5 min at 8,000 rpm, and resuspended in 400 μl 0.1 M potassium phosphate buffer. Aliquots (corresponding to an OD_600_ of 2) (40 μl) of cells expressing kAE1 or the negative control were mixed with 160 μl 0.1 M potassium phosphate buffer and placed into black 96-well plates (Nunc F96; Thermo Scientific), and fluorescence was measured by the use of a fluorometer (Safire2; Tecan). For preparation of a calibration curve, 40-μl aliquots (corresponding to an OD_600_ of 2) were also taken from the 400-μl cultures, centrifuged at 8,000 rpm for 5 min, and washed two times with the different calibration buffers (0.1 M potassium phosphate buffer) containing various adjusted pH values. To prepare the different calibration buffers, certain volumes of 1.0 M KH_2_PO_4_ and 1.0 M K_2_HPO_4_ were initially mixed (94.8 ml of 1.0 M KH_2_PO_4_ and 5.2 ml of 1.0 M K_2_HPO_4_ at pH 5.6; 72.2 ml of 1.0 M KH_2_PO_4_ and 27.8 ml of 1.0 M K_2_HPO_4_ at pH 6.4; 28.3 ml of 1.0 M KH_2_PO_4_ and 71.7 ml of 1.0 M K_2_HPO_4_ at pH 7.2). Then, each of the solutions was adjusted to the desired final pH with H_3_PO_4_/KOH and added to 900 ml distilled H_2_O. Samples were incubated in 200 μl of the corresponding calibration buffers supplemented with 40 μM Nigericin (Sigma-Aldrich) for 1 h at 30°C and 220 rpm. Nigericin was used as a pore-forming agent for permeabilization of the cells, which results in a homogenous pH environment inside the calibration samples. Finally, the levels of fluorescence of 200-μl aliquots of the different calibration samples were also measured in 96-well plates as described above. The excitation wavelength was set to 543 nm, and dual emission was determined at 580 nm and 640 nm. Afterward, fluorescence emission ratios (640 nm/580 nm) and corresponding mean fluorescence values were calculated for each sample (*n* ≥ 3). Simultaneously, the calibration curve was constructed by plotting the fluorescence ratios of the different pH calibration samples as a function of pH and data were fitted by second-order polynomial regression. Based on the obtained equation, mean fluorescence values were converted into intracellular pH values. Finally, intracellular pH values of kAE1-expressing samples were determined by using the calibration curve. All the samples were protected from light throughout the experiment in order to guarantee the stability of the probe.

### GEV yeast strain construction and cultivation conditions.

To generate strain BY4742-GEV, plasmid pAct1-GEV (49) was digested with EcoRV and transformed into BY4742 cells. After homologous recombination, positive integrated clones were selected on leucine dropout plates and finally transformed with pYES-kAE1^WT^ to obtain the β-estradiol-inducible, kAE1-expressing yeast strain BY4742-GEV [pYES-kAE1^WT^]. From a fresh overnight culture of BY4742-GEV [pYES-kAE1^WT^], 10-ml samples (starting OD_600_ = 1) were cultivated at 220 rpm and 30°C in glucose medium containing different concentrations of β-estradiol ranging from 10 nM to 10 μM. After 18 h, pH measurements were performed to determine the cytosolic pH of the samples.

### Anion exchange chromatography.

To measure the intracellular and extracellular Cl^−^ concentrations, BY4742 cells containing empty vector (pPGK) or vector containing wild-type kAE1 (pPGK-kAE1^WT^) were grown to stationary phase (OD_600_ of 3 to 4). As control, cells were additionally pretreated with 200 μM DIDS dissolved in 10 mM HEPES buffer (pH = 7.4) for 30 min at 30°C and 220 rpm to block kAE1 functionality. Then, aliquots of cells corresponding to an OD_600_ of 200 expressing pPGK or pPGK-kAE1^WT^ in the absence or presence of DIDS (disodium 4,4′-diisothiocyanatostilbene-2,2′-disulfonate) were harvested and incubated in 5 ml of HEPES buffer containing 150 mM NaCl with or without DIDS for 1 h at 30°C and 220 rpm. Afterward, the final cell density (OD_600_) was calculated from each sample and aliquots of each cell suspension were taken for each measurement of intra- and extracellular Cl^−^ concentration.

**(i) Sample preparation for extracellular Cl^−^ measurement.** After 1 h of incubation (*t*_1h_), a 1-ml volume of cell suspension was centrifuged for collecting supernatant and diluted (1:10) in MilliQ water. Then, 2 μl from each sample was analyzed via anion-exchange chromatography (Dionex Integrion HPIC system incorporated with a Dionex IonPacTM AS9-HC anion-exchange column) (Thermo Scientific) (2 by 250 mm). As an eluent, 9.0 mM sodium carbonate was used.

**(ii) Sample preparation for intracellular Cl^−^ measurement.** To measure intracellular Cl^−^ concentrations ([Cl^−^]_in_), 1 ml of cell suspension was centrifuged, washed two times rapidly with wash buffer (10 mM Na-HEPES [pH 7.4], 100 μM DIDS, 150 mM sodium gluconate), and resuspended into 1 ml of 10 mM HEPES buffer. Next, cells were lysed in a homogenizer (Precellys Evolution; Bertin Corp.) and supernatant was collected by removing cells debris by centrifugation at high speed. Finally, 10-μl volumes of the collected supernatants were analyzed via anion-exchange chromatography.

**(iii) Calculation of Cl^−^ influx as well as intracellular Cl^−^ concentration.** The Cl^−^ concentration of each sample was calculated from a chloride standard curve (concentrations, 0 mM, 50 mM, 100 mM, 150 mM, and 200 mM); thereby, the measured peak areas of the samples were plotted against the peak areas of the chloride standards. For determination of the total amount of Cl^−^ influx, we additionally measured the extracellular chloride concentration at the hour zero time point (*t*_0h_) and at *t*_1h_ and calculated the total amount of Cl^−^ influx as follows: [Cl^−^]_ex_(*t*_0h_) − [Cl^−^]_ex_(*t*_1h_). Then, the values were normalized to the total cell number (OD_600_ = 1 was set to ∼1 × 10^7^ cells) and illustrated as Cl^−^ influx per cell per hour. For intracellular chloride measurements, the total intracellular Cl^−^ concentration ([Cl^−^]_in_) and OD_600_ were calculated from the 1-ml aliquots and the intracellular chloride concentration was calculated for a single cell.

### Data analysis and statistics.

Statistical analysis was carried out in GraphPad Prism8. All pooled data were determined as mean values ± standard errors of the means (SEM), and statistical significance was assessed by one-way analysis of variance (ANOVA) based on biological replicates and at sample sizes of *n* = ≥3 (*, *P* < 0.05; **, *P* < 0.01; ***, *P* < 0.001).
