# Supplementary figures and images for: Saccharomyces cerevisiae: First Steps to a Suitable Model System To Study the Function and Intracellular Transport of Human Kidney Anion Exchanger 1
Source: mSphere. 2020 Jan 29;5(1):e00802-19. doi: 10.1128/mSphere.00802-19 (PMC6992373; doi:10.1128/mSphere.00802-19)

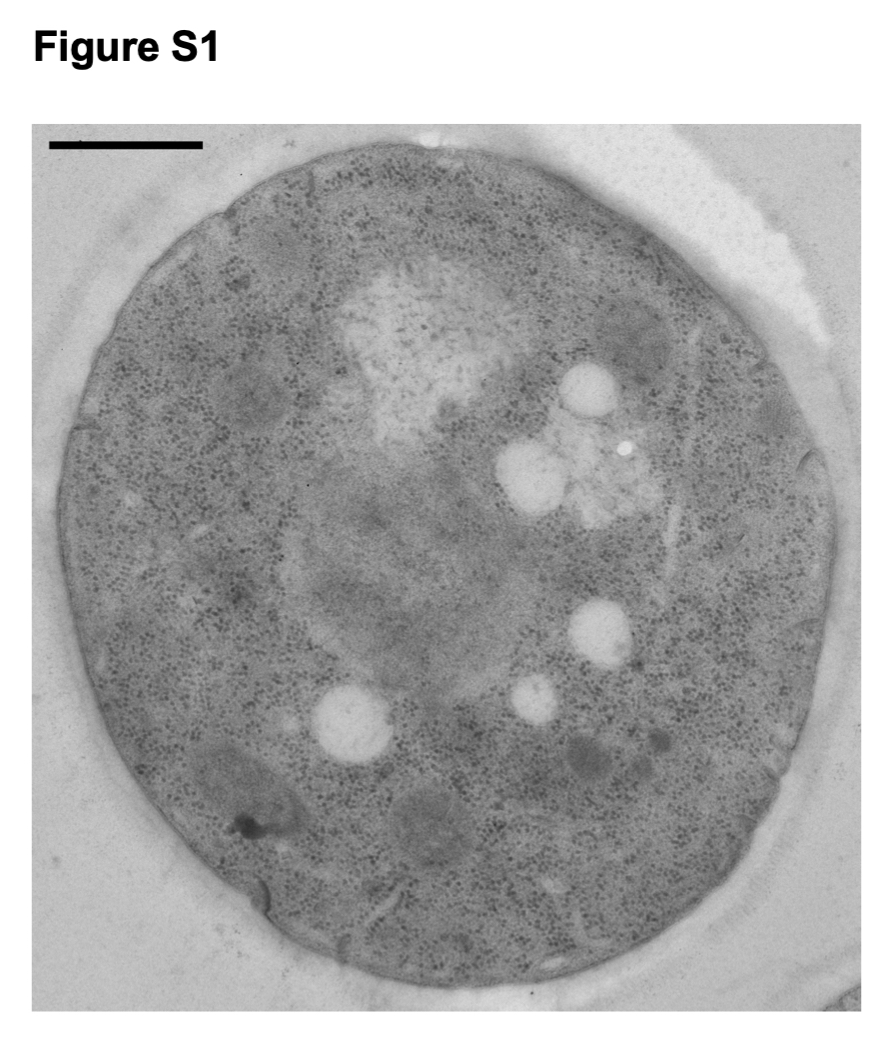

Supplement: FIG S1 [file mSphere.00802-19-sf001.jpg]

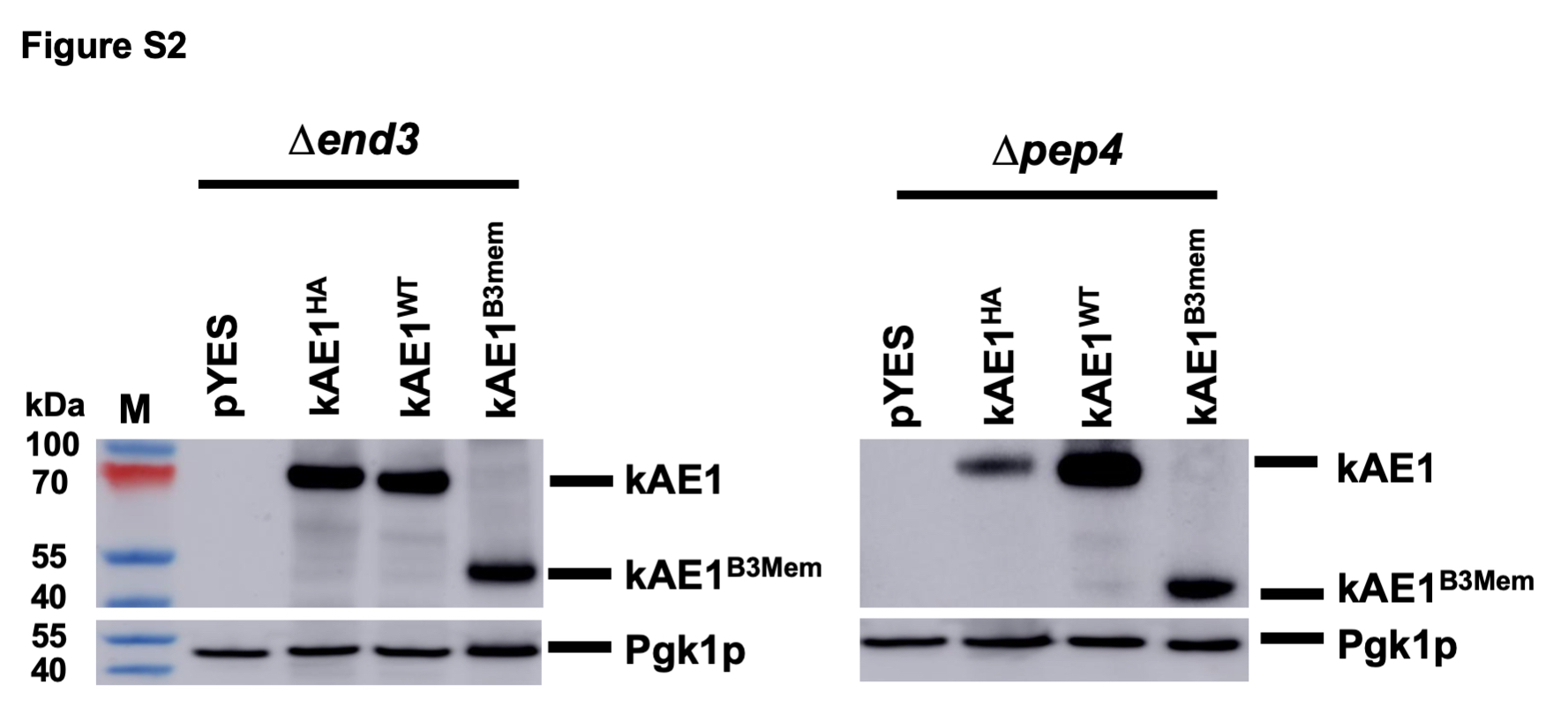

Supplement: FIG S2 [file mSphere.00802-19-sf002.jpg]

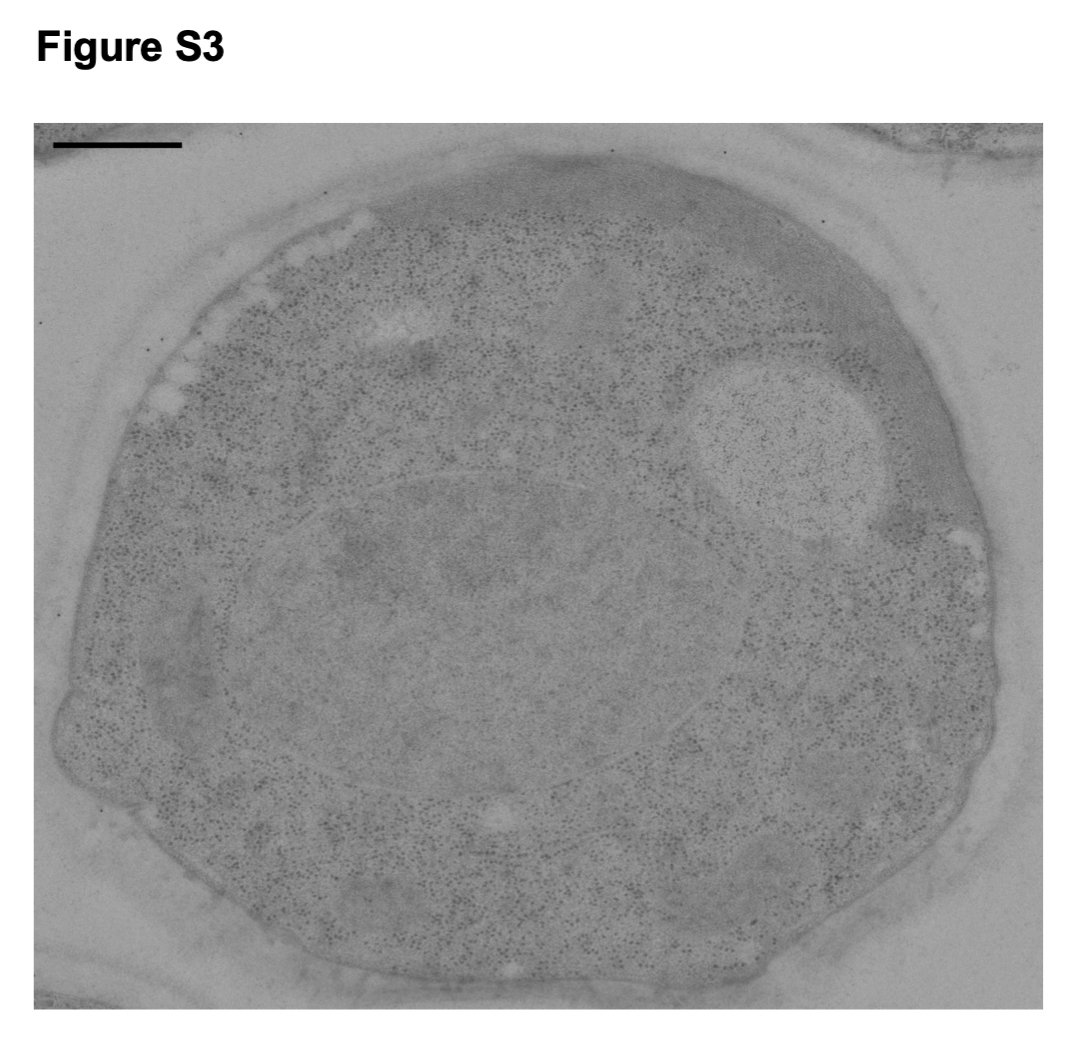

Supplement: FIG S3 [file mSphere.00802-19-sf003.jpg]

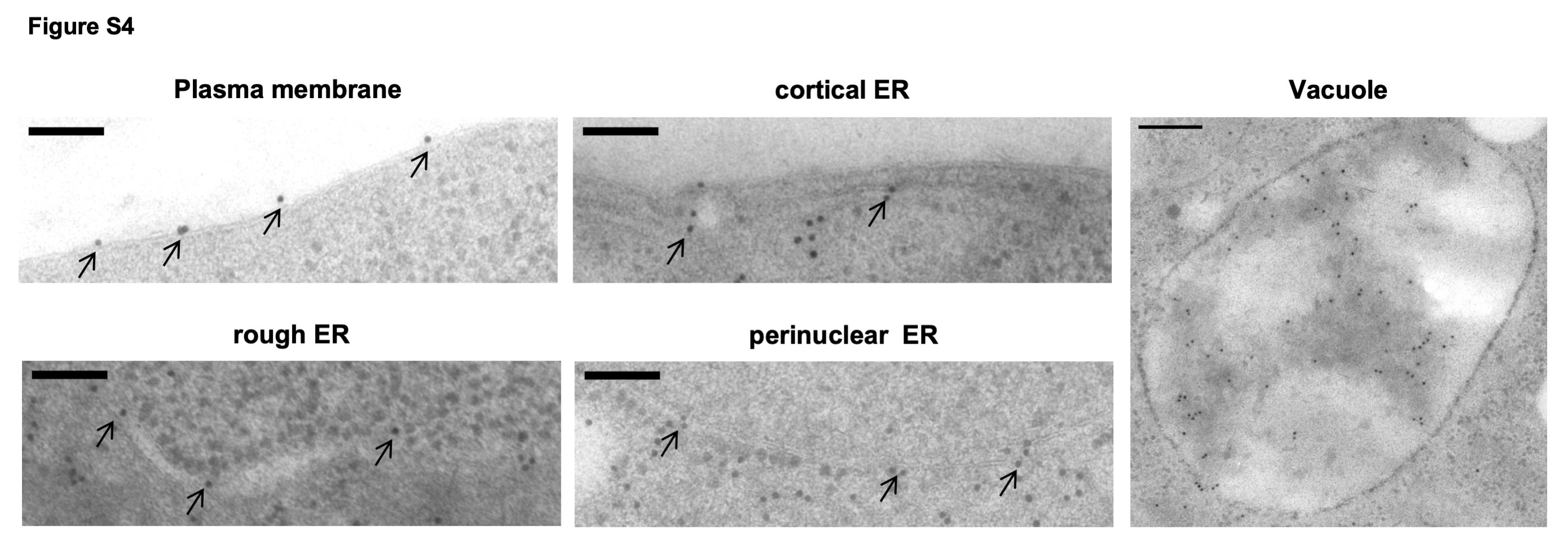

Supplement: FIG S4 [file mSphere.00802-19-sf004.jpg]

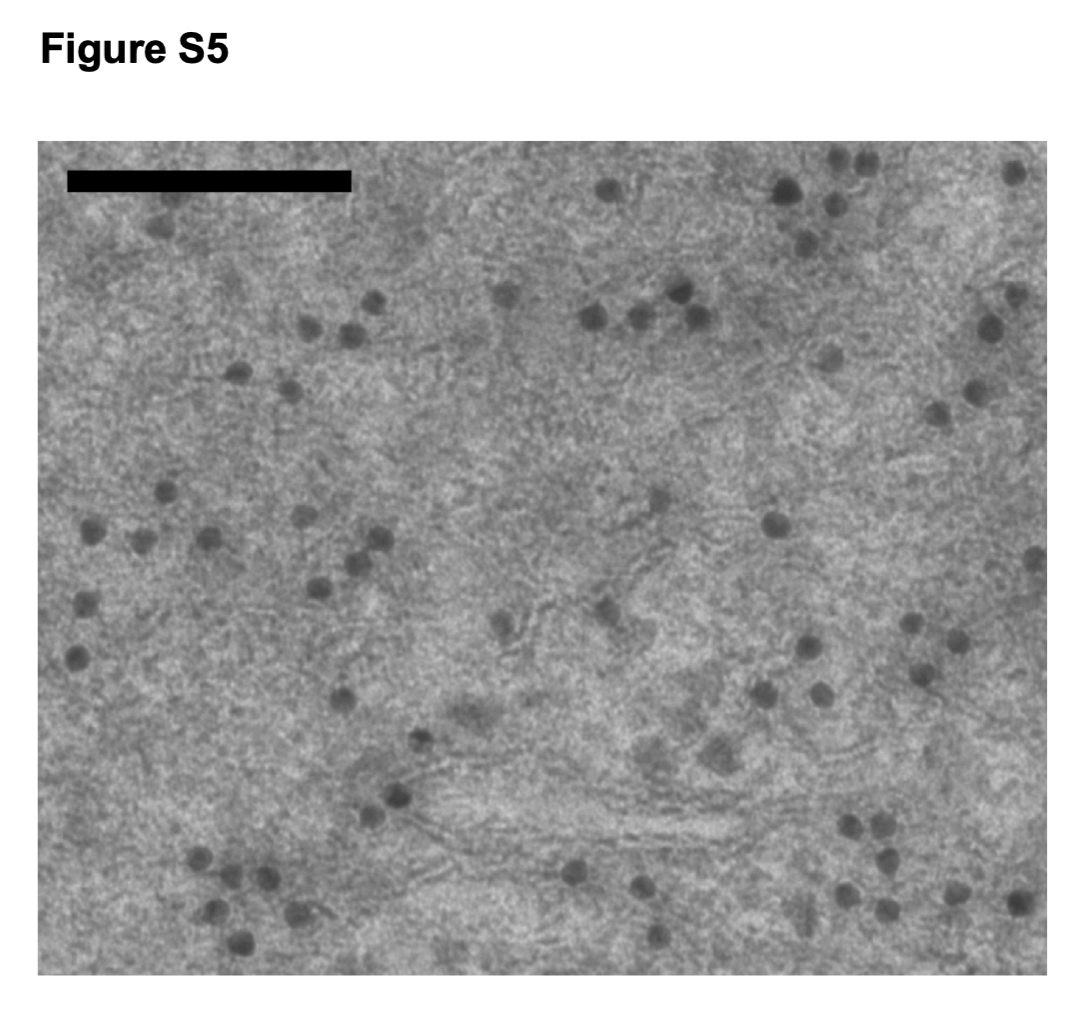

Supplement: FIG S5 [file mSphere.00802-19-sf005.jpg]

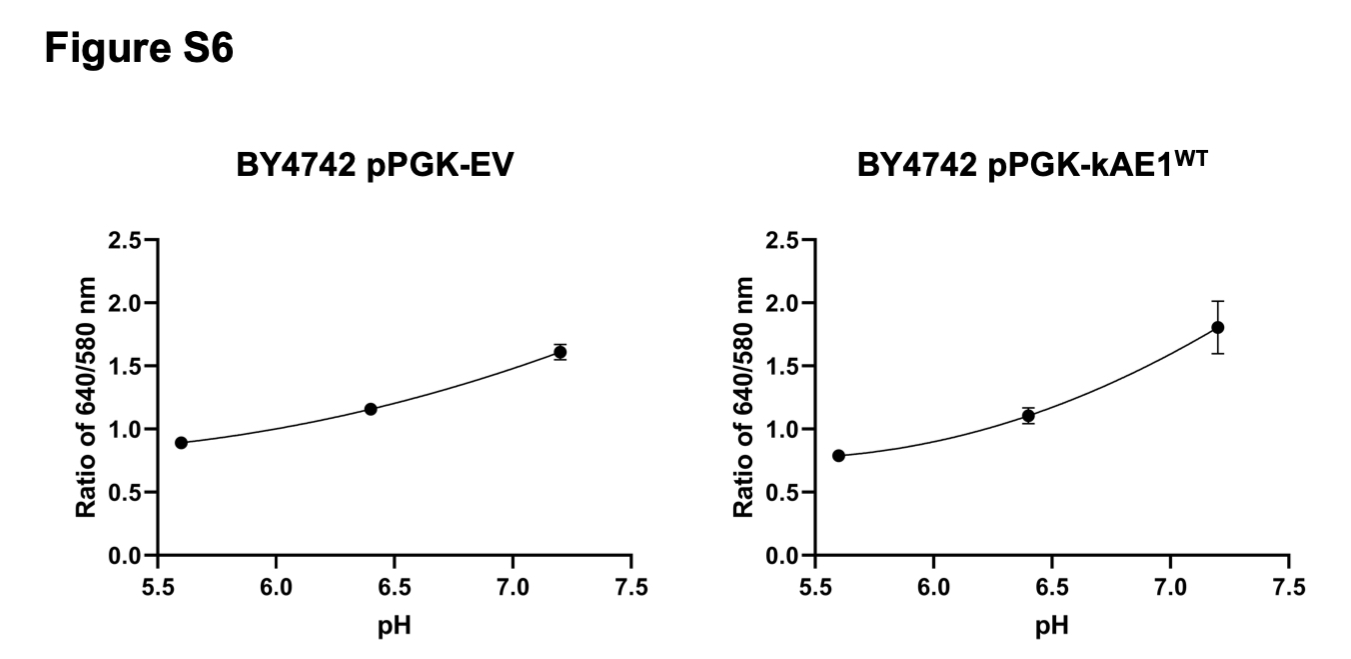

Supplement: FIG S6 [file mSphere.00802-19-sf006.jpg]
