# Supplementary material for: Saccharomyces cerevisiae: First Steps to a Suitable Model System To Study the Function and Intracellular Transport of Human Kidney Anion Exchanger 1
Source: mSphere. 2020 Jan 29;5(1):e00802-19. doi: 10.1128/mSphere.00802-19 (PMC6992373; doi:10.1128/mSphere.00802-19)
Supplement: TABLE S1 [file mSphere.00802-19-st001.pdf]

Table S1.

S. cerevisiae **strain**	**Genotype**	**Reference**
BY4742	MATα *his3Δ1 leu2Δ0 lys2Δ0 ura3Δ0*	Open Biosystems
Δend3	MAT*α end3 his3Δ1 leu2Δ0 lys2Δ0 ura3Δ0*	Open Biosystems
Δpep4	MAT*α pep4 his3Δ1 leu2Δ0 lys2Δ0 ura3Δ0*	Open Biosystems
BY4742 *Pma1-mRFP* + $kAE1^{HA}$	MAT*α his3Δ1 leu2Δ0**:: PMA1-mRFP-LEU2*** *lys2Δ0 ura3Δ0* **[pYES2.1** $\mathbf{kAE1^{HA}}$**]**	This study
BY4742 *Pma1-mRFP* + *yeGFP-kAE1*	MATα *his3Δ1 leu2Δ0**:: PMA1-mRFP-LEU2*** *lys2Δ0 ura3Δ0* **[pYES2.1** ***yeGFP-kAE1***]	This study
Δend3 pYES	MAT*α end3 his3Δ1 leu2Δ0 lys2Δ0 ura3Δ0* [**pYES** ***empty vector***]	This study
Δend3 $kAE1^{HA}$	MAT*α end3 his3Δ1 leu2Δ0 lys2Δ0 ura3Δ0* [**pYES** $\boldsymbol{kAE1^{HA}}$]	This study
Δend3 $kAE1^{WT}$	MAT*α end3 his3Δ1 leu2Δ0 lys2Δ0 ura3Δ0* [**pYES** $\boldsymbol{kAE1^{WT}}$]	This study
Δend3 $kAE1^{B3Mem}$	MAT*α end3 his3Δ1 leu2Δ0 lys2Δ0 ura3Δ0* [**pYES** $\boldsymbol{kAE1^{B3Mem}}$]	This study
Δpep4 pYES	MAT*α pep4 his3Δ1 leu2Δ0 lys2Δ0 ura3Δ0* [**pYES** ***empty vector***]	This study
*Δpep4*$kAE1^{HA}$	MAT*α pep4 his3Δ1 leu2Δ0 lys2Δ0 ura3Δ0* [**pYES** $\boldsymbol{kAE1^{HA}}$]	This study
Δpep4 $kAE1^{WT}$	MAT*α pep4 his3Δ1 leu2Δ0 lys2Δ0 ura3Δ0* [**pYES** $\boldsymbol{kAE1^{WT}}$]	This study
Δpep4 $kAE1^{B3Mem}$	MAT*α pep4 his3Δ1 leu2Δ0 lys2Δ0 ura3Δ0* [**pYES** $\boldsymbol{kAE1^{B3Mem}}$]	This study
BY4742-GEV	MATα *his3Δ1 leu2Δ0 lys2Δ0 ura3Δ0,* leu2Δ0::PACT1-GEV-NatMX	This study
BY4742-GEV pYES-$kAE1^{WT}$	MATα *his3Δ1 leu2Δ0 lys2Δ0 ura3Δ0,* leu2Δ0::PACT1-GEV-NatMX **[pYES2.1** $\mathbf{kAE1^{WT}}$**]**	This study
