# Supplementary material for: Saccharomyces cerevisiae: First Steps to a Suitable Model System To Study the Function and Intracellular Transport of Human Kidney Anion Exchanger 1
Source: mSphere. 2020 Jan 29;5(1):e00802-19. doi: 10.1128/mSphere.00802-19 (PMC6992373; doi:10.1128/mSphere.00802-19)
Supplement: DATA SET S1 [file mSphere.00802-19-sd001.pdf]

Data set S1:

(A) hkAE1^{V5}

ctcgaggaattcATGGACGAAAAGAACCAGGAGCTGAGATGGATGGAGGCGGCGCGCTGGGTGCAACTGGAGGAGAACC
TGGGGGAGAATGGGGCCTGGGGCCGCCCGCACCTCTCTCACCTCACCTTCTGGAGCCTCCTAGAGCTGCGTAGAGT
CTTCACCAAGGGTACTGTCCTCCTAGACCTGCAAGAGACCTCCCTGGCTGGAGTGGCCAACCAACTGCTAGACAGG
TTTATCTTTGAAGACCAGATCCGGCCTCAGGACCGAGAGGAGCTGCTCCGGGCCCTGCTGCTTAAACACAGCCACG
CTGGAGAGCTGGAGGCCCTGGGGGGTGTGAAGCCTGCAGTCCTGACACGCTCTGGTGATCCTTCACAGCCTCTGCT
CCCCCAACACTCCTCACTGGAGACACAGCTCTTCTGTGAGCAGGGAGATGGGGGCACAGAAGGGCACTCACCATC
TGGTATTCTGGAAAAGATTCCCCCGGATTCAGAGGCCACGTTGGTGCTAGTGGGCCGCGCCGACTTCCTGGAGCAG
CCGGTGCTGGGCTTCGTGAGGCTGCAGGAGGCAGCGGAGCTGGAGGCGGTGGAGCTGCCGGTGCCTATACGCTTC
CTCTTTGTGTTGCTGGGACCTGAGGCCCCCCACATCGATTACACCCAGCTTGGCCGGGCTGCTGCCACCCTCATGTC
AGAGAGGGTGTTCCGCATAGATGCCTACATGGCTCAGAGCCGAGGGGAGCTGCTGCACTCCCTAGAGGGCTTCCT
GGACTGCAGCCTAGTGCTGCCTCCCACCGATGCCCCCTCCGAGCAGGCACTGCTCAGTCTGGTGCCTGTGCAGAGG
GAGCTACTTCGAAGGCGCTATCAGTCCAGCCCTGCCAAGCCAGACTCCAGCTTCTACAAGGGCCTAGACTTAAATG
GGGGCCCAGATGACCCTCTGCAGCAGACAGGCCAGCTCTTCGGGGGCCTGGTGCGTGATATCCGGCGCCGCTACC
CCTATTACCTGAGTGACATCACAGATGCATTCAGCCCCCAGGTCCTGGCTGCCGTCATCTTCATCTACTTTGCTGCA
CTGTCACCCGCCATCACCTTCGGCGGCCTCCTGGGAGAAAAGACCCGGAACCAGATGGGAGTGTCGGAGCTGCTG
ATCTCCACTGCAGTGCAGGGCATTCTCTTCGCCCTGCTGGGGGCTCAGCCCCTGCTTGTGGTCGGCTTCTCAGGACC
CCTGCTGGTGTTTGAGGAAGCCTTCTTCTCGTTCTGCGAGACCAACGGTCTAGAGTACATCGTGGGCCGCGTGTGG
ATCGGCTTCTGGCTCATCCTGCTGGTGGTGTTGGTGGTGGCCTTCGAGGGTAGCTTCCTGGTCCGCTTCATCTCCCG
CTATACCCAGGAGATCTTCTCCTTCCTCATTTCCCTCATCTTCATCTATGAGACTTTCTCCAAGCTGATCAAGATCTT
CCAGGACCACCCACTACAGAAGACTTATAACTACAACGTGTTGATGGTGCCCAAACCTCAGGGCCCCCTGCCCAA
CACAGCCCTCCTCTCCCTTGTGCTCATGGCCGGTACCTTCTTCTTTGCCATGATGCTGCGCAAGTTCAAGAACAGCT
CCTATTTCCCTGGCAAGCTGCGTCGGGTCATCGGGGACTTCGGGGTCCCCATCTCCATCCTGATCATGGTCCTGGTG
GATTTCTTCATTCAGGATACCTACACCCAGAAACTCTCGGTGCCTGATGGCTTCAAGGTGTCCAACTCCTCAGCCC
GGGGCTGGGTCATCCACCCACTGGGCTTGCGTTCCGAGTTTCCCATCTGGATGATGTTTGCCTCCGCCCTGCCTGCT
CTGCTGGTCTTCATCCTCATATTCCTGGAGTCTCAGATCACCACGCTGATTGTCAGCAAACCTGAGCGCAAGATGG
TCAAGGGCTCCGGCTTCCACCTGGACCTGCTGCTGGTAGTAGGCATGGGTGGGGTGGCCGCCCTCTTTGGGATGCC
CTGGCTCAGTGCCACCACCGTGCGTTCCGTCACCCATGCCAACGCCCTCACTGTCATGGGCAAAGCCAGCACCCCA
GGGGCTGCAGCCCAGATCCAGGAGGTCAAAGAGCAGCGGATCAGTGGACTCCTGGTCGCTGTGCTTGTGGGCCTG
TCCATCCTCATGGAGCCCATCCTGTCCCGCATCCCCCTGGCTGTACTGTTTGGCATCTTCCTCTACATGGGGGTCAC
GTCGCTCAGCGGCATCCAGCTCTTTGACCGCATCTTGCTTCTGTTCAAGCCACCCAAGTATCACCCAGATGTGCCCT
ACGTCAAGCGGGTGAAGACCTGGCGCATGCACTTATTCACGGGCATCCAGATCATCTGCCTGGCAGTGCTGTGGGT
GGTGAAGTCCACGCCGGCCTCCCTGGCCCTGCCCTTCGTCCTCATCCTCACTGTGCCGCTGCGGCGCGTCCTGCTGC
CGCTCATCTTCAGGAACGTGGAGCTTCAGTGTCTGGATGCTGATGATGCCAAGGCAACCTTTGATGAGGAGGAAG
GTCGGGATGAATACGACGAAGTGGCCATGCCTGTG**GGTAAGCCTATCCCTAACCCTCTCCTCGGTCTCGATTCT**
ACGTGAggatccgtcgac

(B) Kar2^{SS}-hkAE1^{V5}

ctcgaggaattcATGTTTTTCAACAGACTAAGCGCTGGCAAGCTGCTGGTACCACTCTCCGTGGTCCTGTACGCCCTTTTC
GTGGTAATATTACCTTTACAGAATTCTTTCCACTCCTCCAATGTTTTAGTTAGAGGTGCCGATGACGAAAAGAACC
AGGAGCTGAGATGGATGGAGGCGGCGCGCTGGGTGCAACTGGAGGAGAACCTGGGGGAGAATGGGGCCTGGGGC
CGCCCGCACCTCTCTCACCTCACCTTCTGGAGCCTCCTAGAGCTGCGTAGAGTCTTCACCAAGGGTACTGTCCTCCT
AGACCTGCAAGAGACCTCCCTGGCTGGAGTGGCCAACCAACTGCTAGACAGGTTTATCTTTGAAGACCAGATCCG
GCCTCAGGACCGAGAGGAGCTGCTCCGGGCCCTGCTGCTTAAACACAGCCACGCTGGAGAGCTGGAGGCCCTGGG
GGGTGTGAAGCCTGCAGTCCTGACACGCTCTGGTGATCCTTCACAGCCTCTGCTCCCCCAACACTCCTCACTGGAG
ACACAGCTCTTCTGTGAGCAGGGAGATGGGGGCACAGAAGGGCACTCACCATCTGGTATTCTGGAAAAGATTCCC
CCGGATTCAGAGGCCACGTTGGTGCTAGTGGGCCGCGCCGACTTCCTGGAGCAGCCGGTGCTGGGCTTCGTGAGG
CTGCAGGAGGCAGCGGAGCTGGAGGCGGTGGAGCTGCCGGTGCCTATACGCTTCCTCTTTGTGTTGCTGGGACCTG
AGGCCCCCCACATCGATTACACCCAGCTTGGCCGGGCTGCTGCCACCCTCATGTCAGAGAGGGTGTTCCGCATAGA
TGCCTACATGGCTCAGAGCCGAGGGGAGCTGCTGCACTCCCTAGAGGGCTTCCTGGACTGCAGCCTAGTGCTGCCT
CCCACCGATGCCCCCTCCGAGCAGGCACTGCTCAGTCTGGTGCCTGTGCAGAGGGAGCTACTTCGAAGGCGCTATC
AGTCCAGCCCTGCCAAGCCAGACTCCAGCTTCTACAAGGGCCTAGACTTAAATGGGGGCCCAGATGACCCTCTGC
AGCAGACAGGCCAGCTCTTCGGGGGCCTGGTGCGTGATATCCGGCGCCGCTACCCCTATTACCTGAGTGACATCAC
AGATGCATTCAGCCCCCAGGTCCTGGCTGCCGTCATCTTCATCTACTTTGCTGCACTGTCACCCGCCATCACCTTCG
GCGGCCTCCTGGGAGAAAAGACCCGGAACCAGATGGGAGTGTCGGAGCTGCTGATCTCCACTGCAGTGCAGGGCA
TTCTCTTCGCCCTGCTGGGGGCTCAGCCCCTGCTTGTGGTCGGCTTCTCAGGACCCCTGCTGGTGTTTGAGGAAGCC

TTCTTCTCGTTCTGCGAGACCAACGGTCTAGAGTACATCGTGGGCCGCGTGTGGATCGGCTTCTGGCTCATCCTGCT
GGTGGTGTTGGTGGTGGCCTTCGAGGGTAGCTTCCTGGTCCGCTTCATCTCCCGCTATACCCAGGAGATCTTCTCCT
TCCTCATTTCCCTCATCTTCATCTATGAGACTTTCTCCAAGCTGATCAAGATCTTCCAGGACCACCCACTACAGAAG
ACTTATAACTACAACGTGTTGATGGTGCCCAAACCTCAGGGCCCCCTGCCCAACACAGCCCTCCTCTCCCTTGTGCT
CATGGCCGGTACCTTCTTCTTTGCCATGATGCTGCGCAAGTTCAAGAACAGCTCCTATTTCCCTGGCAAGCTGCGTC
GGGTCATCGGGGACTTCGGGGTCCCCATCTCCATCCTGATCATGGTCCTGGTGGATTTCTTCATTCAGGATACCTAC
ACCCAGAAACTCTCGGTGCCTGATGGCTTCAAGGTGTCCAACTCCTCAGCCCGGGGCTGGGTCATCCACCCACTGG
GCTTGCGTTCCGAGTTTCCCATCTGGATGATGTTTGCCTCCGCCCTGCCTGCTCTGCTGGTCTTCATCCTCATATTCC
TGGAGTCTCAGATCACCACGCTGATTGTCAGCAAACCTGAGCGCAAGATGGTCAAGGGCTCCGGCTTCCACCTGG
ACCTGCTGCTGGTAGTAGGCATGGGTGGGGTGGCCGCCCTCTTTGGGATGCCCTGGCTCAGTGCCACCACCGTGCG
TTCCGTCACCCATGCCAACGCCCTCACTGTCATGGGCAAAGCCAGCACCCCAGGGGCTGCAGCCCAGATCCAGGA
GGTCAAAGAGCAGCGGATCAGTGGACTCCTGGTCGCTGTGCTTGTGGGCCTGTCCATCCTCATGGAGCCCATCCTG
TCCCGCATCCCCCTGGCTGTACTGTTTGGCATCTTCCTCTACATGGGGGTCACGTCGCTCAGCGGCATCCAGCTCTT
TGACCGCATCTTGCTTCTGTTCAAGCCACCCAAGTATCACCCAGATGTGCCCTACGTCAAGCGGGTGAAGACCTGG
CGCATGCACTTATTCACGGGCATCCAGATCATCTGCCTGGCAGTGCTGTGGGTGGTGAAGTCCACGCCGGCCTCCC
TGGCCCTGCCCTTCGTCCTCATCCTCACTGTGCCGCTGCGGCGCGTCCTGCTGCCGCTCATCTTCAGGAACGTGGAG
CTTCAGTGTCTGGATGCTGATGATGCCAAGGCAACCTTTGATGAGGAGGAAGGTCGGGATGAATACGACGAAGTG
GCCATGCCTGTG**GGTAAGCCTATCCCTAACCCTCTCCTCGGTCTCGATTCTACG**TGAggatccgtcgac

(C) ykAE1^{V5}

ctcgaggaattcATGGACGAAAAGAATCAAGAATTGAGATGGATGGAAGCTGCTAGATGGGTTCAATTGGAAGAAAATT
TGGGTGAAAATGGTGCTTGGGGTAGACCACATTTGTCTCATTTGACTTTTTGGTCCTTGTTGGAATTGAGAAGAGTT
TTCACTAAGGGTACTGTCTTGTTGGACTTGCAAGAAACTTCTTTGGCTGGTGTTGCCAATCAATTATTGGACAGATT
CATTTTCGAAGATCAAATCAGACCACAAGATAGAGAAGAATTATTGAGAGCCTTGTTGTTGAAGCACTCTCATGCT
GGTGAATTGGAAGCTTTAGGTGGTGTTAAGCCAGCTGTTTTGACTAGATCTGGTGATCCATCACAACCATTATTGC
CACAACATTCTTCATTGGAAACCCAATTATTCTGCGAACAAGGTGATGGTGGTACTGAAGGTCATTCTCCATCTGG
TATTTTGGAAAAGATTCCACCAGATTCTGAAGCCACTTTGGTTTTGGTTGGTAGAGCTGATTTTTTGGAACAACCAG
TTTTGGGTTTCGTTAGATTGCAAGAAGCTGCTGAATTAGAAGCAGTTGAATTGCCAGTTCCAATCAGATTCTTGTTC
GTTTTGTTGGGTCCAGAAGCTCCACATATTGATTATACTCAATTGGGTAGAGCCGCTGCTACTTTGATGTCTGAAAG
AGTTTTTAGAATCGACGCTTACATGGCTCAATCCAGAGGTGAATTATTGCATTCTTTGGAAGGTTTCTTGGACTGCT
CTTTAGTTTTGCCACCAACTGATGCTCCATCTGAACAAGCTTTGTTGTCTTTGGTTCCAGTTCAAAGAGAATTATTG
AGAAGAAGATACCAATCCTCTCCAGCTAAACCAGACTCTTCATTTTACAAGGGTTTGGATTTGAATGGTGGTCCAG
ATGATCCATTGCAACAAACTGGTCAATTATTCGGTGGTTTGGTTAGAGACATCAGAAGAAGATATCCTTACTACTT
GTCCGATATCACCGATGCTTTTTCACCACAAGTTTTGGCTGCTGTTATCTTCATCTATTTCGCTGCTTTGTCACCAGC
TATTACTTTTGGTGGTTTATTAGGTGAAAAGACCAGAAATCAAATGGGTGTTTCCGAATTATTGATCTCCACTGCTG
TTCAAGGTATTTTGTTTGCTTTGTTAGGTGCCCAACCTTTGTTGGTTGTTGGTTTTTCTGGTCCATTATTGGTCTTTGA
AGAAGCTTTCTTCTCCTTCTGTGAAACTAACGGTTTGGAATATATCGTCGGTAGAGTTTGGATTGGTTTCTGGTTAA
TTTTGTTGGTCGTTTTGGTCGTCGCTTTCGAAGGTTCATTTTGGTAAGATTCATCTCCAGATACACCCAAGAAATC
TTTTCTTTCTTGATCTCCTTGATTTTCATCTACGAAACCTTCTCCAAGTTGATCAAGATCTTCCAAGATCACCCATTG
CAAAAGACCTACAACTACAACGTTTTGATGGTTCCAAAACCACAAGGTCCATTGCCAAATACTGCTTTATTATCCT
TGGTTTTAATGGCCGGTACTTTCTTCTTTGCCATGATGTTGAGAAAGTTCAAGAACTCCTCTTACTTCCCAGGTAAG
TTGAGAAGAGTCATTGGTGATTTTGGTGTCCCAATCTCCATTTTGATTATGGTCTTGGTTGACTTCTTCATCCAAGA
TACTTACACCCAAAAGTTGTCTGTTCCAGATGGTTTCAAGGTCAGTAATTCTTCAGCTAGAGGTTGGGTTATTCATC
CATTGGGTTTGAGATCCGAATTTCCAATTTGGATGATGTTCGCTTCTGCTTTGCCAGCTTTATTGGTTTTTATCTTGA
TATTCTTGGAATCCCAAATCACCACCTTGATCGTTTCTAAACCTGAAAGAAAGATGGTCAAGGGTTCTGGTTTTCAC
TTGGATTTGTTGTTAGTTGTCGGTATGGGTGGTGTTGCAGCTTTGTTTGGTATGCCATGGTTGTCTGCTACTACTGTT
AGATCTGTTACTCATGCTAACGCTTTGACTGTTATGGGTAAAGCTTCTACTCCAGGTGCTGCTGCTCAAATTCAAGA
AGTAAAAGAACAAAGAATTTCCGGTTTGTTGGTAGCCGTTTTAGTTGGTTTGTCAATCTTGATGGAACCTATCTTGT
CTAGAATCCCATTGGCTGTTTTGTTCGGTATTTTCTTGTACATGGGTGTCACATCCTTGTCCGGTATCCAATTATTTG
ATAGAATCTTGTTGTTATTCAAGCCACCAAAGTACCATCCAGATGTTCCTTATGTTAAGAGAGTCAAGACTTGGAG
AATGCATTTGTTCACCGGTATTCAAATTATCTGCTTGGCAGTTTTGTGGGTTGTTAAGTCAACTCCAGCTTCTTTAG
CATTGCCATTCGTTTTGATTTTGACCGTCCCATTAAGAAGAGTCTTGTTGCCATTGATATTCAGAAACGTCGAATTG
CAATGTTTGGATGCTGATGATGCTAAGGCTACTTTTGACGAAGAAGAAGGTAGAGACGAATACGATGAAGTTGCT
ATGCCAGTT**GGTAAGCCAATTCCAAATCCATTATTAGGTTTGGACTCCACT**TAAggatccgtcgac

(D) kAE1^{HA}

ctcgagATGGACGAAAAGAATCAAGAATTGAGATGGATGGAAGCTGCTAGATGGGTTCAATTGGAAGAAAATTTGGG
TGAAAATGGTGCTTGGGGTAGACCACATTTGTCTCATTTGACTTTTTGGTCCTTGTTGGAATTGAGAAGAGTTTTCA

CTAAGGGTACTGTCTTGTTGGACTTGCAAGAAACTTCTTTGGCTGGTGTTGCCAATCAATTATTGGACAGATTCATT
TTCGAAGATCAAATCAGACCACAAGATAGAGAAGAATTATTGAGAGCCTTGTTGTTGAAGCACTCTCATGCTGGTG
AATTGGAAGCTTTAGGTGGTGTTAAGCCAGCTGTTTTGACTAGATCTGGTGATCCATCACAACCATTATTGCCACA
ACATTCTTCATTGGAAACCCAATTATTCTGCGAACAAGGTGATGGTGGTACTGAAGGTCATTCTCCATCTGGTATTT
TGGAAAAGATTCCACCAGATTCTGAAGCCACTTTGGTTTTGGTTGGTAGAGCTGATTTTTGGAACAACCAGTTTTG
GGTTTCGTTAGATTGCAAGAAGCTGCTGAATTAGAAGCAGTTGAATTGCCAGTTCCAATCAGATTCTTGTTCGTTTT
GTTGGGTCCAGAAGCTCCACATATTGATTATACTCAATTGGGTAGAGCCGCTGCTACTTTGATGTCTGAAAGAGTT
TTTAGAATCGACGCTTACATGGCTCAATCCAGAGGTGAATTATTGCATTCTTTGGAAGGTTTCTTGGACTGCTCTTT
AGTTTTGCCACCAACTGATGCTCCATCTGAACAAGCTTTGTTGTCTTTGGTTCCAGTTCAAAGAGAATTATTGAGAA
GAAGATACCAATCCTCTCCAGCTAAACCAGACTCTTCATTTTACAAGGGTTTGGATTTGAATGGTGGTCCAGATGA
TCCATTGCAACAAACTGGTCAATTATTCGGTGGTTTGGTTAGAGACATCAGAAGAAGATATCCTTACTACTTGTCC
GATATCACCGATGCTTTTTCACCACAAGTTTTGGCTGCTGTTATCTTCATCTATTTCGCTGCTTTGTCACCAGCTATT
ACTTTTGGTGGTTTATTAGGTGAAAAGACCAGAAATCAAATGGGTGTTTCCGAATTATTGATCTCCACTGCTGTTCA
AGGTATTTTGTTTGCTTTGTTAGGTGCCCAACCTTTGTTGGTTGTTGGTTTTTCTGGTCCATTATTGGTCTTTGAAGA
AGCTTTCTTCTCCTTCTGTGAAACTAACGGTTTGGAATATATCGTCGGTAGAGTTTGGATTGGTTTCTGGTTAATTTT
GTTGGTCGTTTTGGTCGTCGCTTTCGAAGGTTCATTTTTGGTAAGATTCATCTCCAGATACACCCAAGAAATCTTTT
CTTTCTTGATCTCCTTGATTTTCATCTACGAAACCTTCTCCAAGTTGATCAAGATCTTCCAAGATCACCCATTGCAA
AAGACCTACAACTACAAC**TACCCATACGATGTTCCAGATTACGCT**GTTTTGATGGTTCCAAAACCACAAGGTCC
ATTGCCAAATACTGCTTTATTATCCTTGGTTTTAATGGCCGGTACTTTCTTCTTTGCCATGATGTTGAGAAAGTTCAA
GAACTCCTCTTACTTCCCAGGTAAGTTGAGAAGAGTCATTGGTGATTTTGGTGTCCCAATCTCCATTTTGATTATGG
TCTTGGTTGACTTCTTCATCCAAGATACTTACACCCAAAAGTTGTCTGTTCCAGATGGTTTCAAGGTCAGTAATTCT
TCAGCTAGAGGTTGGGTTATTCATCCATTGGGTTTGAGATCCGAATTTCCAATTTGGATGATGTTCGCTTCTGCTTT
GCCAGCTTTATTGGTTTTTATCTTGATATTCTTGGAATCCCAAATCACCACCTTGATCGTTTCTAAACCTGAAAGAA
AGATGGTCAAGGGTTCTGGTTTTCACTTGGATTTGTTGTTAGTTGTCGGTATGGGTGGTGTTGCAGCTTTGTTTGGT
ATGCCATGGTTGTCTGCTACTACTGTTAGATCTGTTACTCATGCTAACGCTTTGACTGTTATGGGTAAAGCTTCTAC
TCCAGGTGCTGCTGCTCAAATTCAAGAAGTAAAAGAACAAAGAATTTCCGGTTTGTTGGTAGCCGTTTTAGTTGGT
TTGTCAATCTTGATGGAACCTATCTTGTCTAGAATCCCATTGGCTGTTTTGTTCGGTATTTTCTTGTACATGGGTGTC
ACATCCTTGTCCGGTATCCAATTATTTGATAGAATCTTGTTGTTATTCAAGCCACCAAAGTACCATCCAGATGTTCC
TTATGTTAAGAGAGTCAAGACTTGGAGAATGCATTTGTTCACCGGTATTCAAATTATCTGCTTGGCAGTTTTGTGGG
TTGTTAAGTCAACTCCAGCTTCTTTAGCATTGCCATTCGTTTTGATTTTGACCGTCCCATTAAGAAGAGTCTTGTTGC
CATTGATATTCAGAAACGTCGAATTGCAATGTTTGGATGCTGATGATGCTAAGGCTACTTTTGACGAAGAAGAAGG
TAGAGACGAATACGATGAAGTTGCTATGCCAGTTTAAggatcc

(E) kAE1^{B3mem} (aa 361 to 911)

gtcgacggattcctcgagATG**TACCCATACGATGTTCCAGATTACGCT**ggtggtggtggtagtGGTTTGGATTTGAATGGTGGTCCA
GATGATCCATTGCAACAAACTGGTCAATTATTCGGTGGTTTGGTTAGAGACATCAGAAGAAGATATCCTTACTACT
TGTCCGATATCACCGATGCTTTTTCACCACAAGTTTTGGCTGCTGTTATCTTCATCTATTTCGCTGCTTTGTCACCAG
CTATTACTTTTGGTGGTTTATTAGGTGAAAAGACCAGAAATCAAATGGGTGTTTCCGAATTATTGATCTCCACTGCT
GTTCAAGGTATTTTGTTTGCTTTGTTAGGTGCCCAACCTTTGTTGGTTGTTGGTTTTTCTGGTCCATTATTGGTCTTTG
AAGAAGCTTTCTTCTCCTTCTGTGAAACTAACGGTTTGGAATATATCGTCGGTAGAGTTTGGATTGGTTTCTGGTTA
ATTTTGTTGGTCGTTTTGGTCGTCGCTTTCGAAGGTTCATTTTTGGTAAGATTCATCTCCAGATACACCCAAGAAAT
CTTTTCTTTCTTGATCTCCTTGATTTTCATCTACGAAACCTTCTCCAAGTTGATCAAGATCTTCCAAGATCACCCATT
GCAAAAGACCTACAACTACAACGTTTTGATGGTTCCAAAACCACAAGGTCCATTGCCAAATACTGCTTTATTATCC
TTGGTTTTAATGGCCGGTACTTTCTTCTTTGCCATGATGTTGAGAAAGTTCAAGAACTCCTCTTACTTCCCAGGTAA
GTTGAGAAGAGTCATTGGTGATTTTGGTGTCCCAATCTCCATTTTGATTATGGTCTTGGTTGACTTCTTCATCCAAG
ATACTTACACCCAAAAGTTGTCTGTTCCAGATGGTTTCAAGGTCAGTAATTCTTCAGCTAGAGGTTGGGTTATTCAT
CCATTGGGTTTGAGATCCGAATTTCCAATTTGGATGATGTTCGCTTCTGCTTTGCCAGCTTTATTGGTTTTTATCTTG
ATATTCTTGGAATCCCAAATCACCACCTTGATCGTTTCTAAACCTGAAAGAAAGATGGTCAAGGGTTCTGGTTTTC
ACTTGGATTTGTTGTTAGTTGTCGGTATGGGTGGTGTTGCAGCTTTGTTTGGTATGCCATGGTTGTCTGCTACTACTG
TTAGATCTGTTACTCATGCTAACGCTTTGACTGTTATGGGTAAAGCTTCTACTCCAGGTGCTGCTGCTCAAATTCAA
GAAGTAAAAGAACAAAGAATTTCCGGTTTGTTGGTAGCCGTTTTAGTTGGTTTGTCAATCTTGATGGAACCTATCTT
GTCTAGAATCCCATTGGCTGTTTTGTTCGGTATTTTCTTGTACATGGGTGTCACATCCTTGTCCGGTATCCAATTATT
TGATAGAATCTTGTTGTTATTCAAGCCACCAAAGTACCATCCAGATGTTCCTTATGTTAAGAGAGTCAAGACTTGG
AGAATGCATTTGTTCACCGGTATTCAAATTATCTGCTTGGCAGTTTTGTGGGTTGTTAAGTCAACTCCAGCTTCTTT
AGCATTGCCATTCGTTTTGATTTTGACCGTCCCATTAAGAAGAGTCTTGTTGCCATTGATATTCAGAAACGTCGAAT
TGCAATGTTTGGATGCTGATGATGCTAAGGCTACTTTTGACGAAGAAGAAGGTAGAGACGAATACGATGAAGTTG
CTATGCCAGTTTAAggatccgagctc
