# Supplementary material for: Saccharomyces cerevisiae: First Steps to a Suitable Model System To Study the Function and Intracellular Transport of Human Kidney Anion Exchanger 1
Source: mSphere. 2020 Jan 29;5(1):e00802-19. doi: 10.1128/mSphere.00802-19 (PMC6992373; doi:10.1128/mSphere.00802-19)
Supplement: TABLE S2 [file mSphere.00802-19-st002.pdf]

Table S2.

Primer	Construct	5'-3' Sequence
5' kAE1	Amplification of untagged wild-type kAE1 with yeast-optimized codon usage	ctcgaggaattcATGGACGAAAAGAATCAAGAATTGAGATG
3' kAE1	Amplification of untagged wild-type kAE1 with yeast-optimized codon usage	gtcgacggatccTTAAACTGGCATAGCAACTTCATCGTATTC
5' yeGFP	Overhang primer for *in vivo* recombination in yeast with pYES vector	CACTATAGGGAATATTAAGCTCGCctcgagATGTCT AAAGGTGAAGAATTATTCAC
3' yeGFP	Overhang primer for *in vivo* recombination in yeast with ykAE1	CATCTCAATTCTTGATTCTTTTCGTCactaccaccaccacc TTTGTACAATTCATCCATACCA
