# Supplementary material for: Saccharomyces cerevisiae: First Steps to a Suitable Model System To Study the Function and Intracellular Transport of Human Kidney Anion Exchanger 1
Source: mSphere. 2020 Jan 29;5(1):e00802-19. doi: 10.1128/mSphere.00802-19 (PMC6992373; doi:10.1128/mSphere.00802-19)
Supplement: TABLE S3 [file mSphere.00802-19-st003.pdf]

Table S3.

Antibody	Dilution	Source
anti-PGK1, mouse	1 : 1,000	Life Technologies (Novex)
anti-Mouse-HRP	1 : 10,000	Sigma
anti-Rabbit-HRP	1 : 10,000	Sigma
anti-GFP, mouse	1 : 1,000	Roche
anti-V5, mouse	1 : 1,000	AbD SeroTec
anti-kAE1, rabbit	1 : 1,000	(1), against N-terminal region of kAE1 (aa 65 to 80)
anti-kAE1, mouse (BRIC170)	1 : 1,000	IBGRL (9450PA), epitope in aa region 368 to 382 is recognized
anti-HA, rat	1 : 40	Roche
anti-rat-FITC	1 : 160	Sigma

References

1. Wu F, Saleem MA, Kampik NB, Satchwell TJ, Williamson RC, Blattner SM, Ni L, Toth T, White G, Young MT, Parker MD, Alper SL, Wagner CA, Toye AM. 2010. Anion exchanger 1 interacts with nephrin in podocytes. J Am Soc Nephrol 21:1456-67.
